# Causal effects of plasma metabolites on chronic kidney diseases and renal function: a bidirectional Mendelian randomization study

**DOI:** 10.3389/fendo.2024.1429159

**Published:** 2024-07-26

**Authors:** Xiaodong Zhao, Jialin Gao, Kai Kou, Xi Wang, Xin Gao, Yishu Wang, Honglan Zhou, Faping Li

**Affiliations:** ^1^ Department of Urology, The First Hospital of Jilin University, Changchun, China; ^2^ Department of Hepatobiliary and Pancreatic Surgery, General Surgery Center, The First Hospital of Jilin University, Changchun, Jilin, China; ^3^ Department of Endocrinology, The Second Affiliated Hospital of Zhengzhou University, Zhengzhou, China; ^4^ Key Laboratory of Pathobiology, Ministry of Education, Jilin University, Changchun, China

**Keywords:** chronic kidney disease, plasma metabolites, Mendelian randomization, renal function, genetic causal association

## Abstract

**Background:**

Despite the potential demonstrated by targeted plasma metabolite modulators in halting the progression of chronic kidney disease (CKD), a lingering uncertainty persists concerning the causal relationship between distinct plasma metabolites and the onset and progression of CKD.

**Methods:**

A genome-wide association study was conducted on 1,091 metabolites and 309 metabolite ratios derived from a cohort of 8,299 unrelated individuals of European descent. Employing a bidirectional two-sample Mendelian randomization (MR) analysis in conjunction with colocalization analysis, we systematically investigated the associations between these metabolites and three phenotypes: CKD, creatinine-estimated glomerular filtration rate (creatinine-eGFR), and urine albumin creatinine ratio (UACR). In the MR analysis, the primary analytical approach employed was inverse variance weighting (IVW), and sensitivity analysis was executed utilizing the MR-Egger method and MR-pleiotropy residual sum and outlier (MR-PRESSO). Heterogeneity was carefully evaluated through Cochrane’s Q test. To ensure the robustness of our MR results, the leave-one-out method was implemented, and the strength of causal relationships was subjected to scrutiny via Bonferroni correction.

**Results:**

Our thorough MR analysis involving 1,400 plasma metabolites and three clinical phenotypes yielded a discerning identification of 21 plasma metabolites significantly associated with diverse outcomes. Specifically, in the forward MR analysis, 6 plasma metabolites were determined to be causally associated with CKD, 16 with creatinine-eGFR, and 7 with UACR. Substantiated by robust evidence from colocalization analysis, 6 plasma metabolites shared causal variants with CKD, 16 with creatinine-eGFR, and 7 with UACR. In the reverse analysis, a diminished creatinine-eGFR was linked to elevated levels of nine plasma metabolites. Notably, no discernible associations were observed between other plasma metabolites and CKD, creatinine-eGFR, and UACR. Importantly, our analysis detected no evidence of horizontal pleiotropy.

**Conclusion:**

This study elucidates specific plasma metabolites causally associated with CKD and renal functions, providing potential targets for intervention. These findings contribute to an enriched understanding of the genetic underpinnings of CKD and renal functions, paving the way for precision medicine applications and therapeutic strategies aimed at impeding disease progression.

## 1 Introduction

Chronic kidney disease (CKD) is distinguished by structural and functional impairments in renal physiology. Diagnosis typically involves an estimated glomerular filtration rate (eGFR) below 60 mL/min per 1.73 m^2^ or elevated indicators of kidney damage persisting for at least three months (1). Globally, CKD affects nearly 10% of the adult population, resulting in an annual toll of 1.2 million deaths and the loss of 28.0 million years of life (2). Projections indicate that by 2040, CKD is poised to become the fifth leading cause of mortality worldwide (3). Given its burgeoning impact, elucidating the determinants of CKD assumes paramount significance for devising targeted primary prevention strategies with substantial public health implications.

In recent years, a burgeoning body of evidence has underscored the close association between metabolic dysregulation and CKD development. Both *in vivo* and *in vitro* investigations have delineated potential links between CKD and metabolic disorders such as hypertension, diabetes mellitus (or insulin resistance), and dyslipidemia (4–7). Hypertension, diabetes mellitus, and dyslipidemia are prominent contributors to CKD progression. These conditions induce chronic inflammation, oxidative stress, and endothelial dysfunction, which damage renal structures and impair their function. Insulin resistance, commonly associated with obesity and diabetes, exacerbates renal damage by promoting glomerular hypertension and hyperfiltration (8). Dyslipidemia leads to lipid accumulation in renal tissues, contributing to tubulointerstitial fibrosis and glomerulosclerosis (9). Furthermore, there are indications in some studies that these disorders may share common pathological pathways (10). The contemporary deployment of OMIC technologies, encompassing genomics and metabolomics, has facilitated the exploration of underlying pathophysiological mechanisms and potential therapeutic avenues for human diseases. Recent metabolomic studies in both human and animal models have identified various circulating biomarkers, including amino acids, phosphates, and lipids (11–13). Emerging evidence suggests that altered gut microbiota and metabolite profiles, such as increased levels of trimethylamine N-oxide (TMAO), may play a role in CKD development by promoting inflammation and vascular dysfunction (14). However, challenges stemming from sample size limitations and confounding factors underscore the uncertainty surrounding the causal effect of plasma metabolites on CKD.

Mendelian randomization (MR), leveraging genetic variants to establish causal relationships, serves as a methodological approach circumventing biases, reminiscent of a large-scale randomized controlled trial (RCT) mirroring natural processes (15). This technique is particularly useful in chronic diseases due to several reasons. Firstly, chronic diseases often develop over a long period, making it difficult to establish causal links through traditional observational studies. MR uses genetic variants, which are fixed at conception and thus precede disease onset, providing a time-anchored method to infer causality (16). Secondly, MR can help identify modifiable risk factors for chronic diseases, aiding in the development of preventive and therapeutic strategies. For instance, MR has been used to demonstrate the causal role of elevated body mass index (BMI) in the development of type 2 diabetes and cardiovascular diseases (17), highlighting the importance of weight management in chronic disease prevention. Thirdly, MR is useful in exploring the biological mechanisms underlying chronic diseases. By linking genetic variants associated with specific biological pathways to disease outcomes, MR can uncover potential therapeutic targets. A recent MR analysis has yielded novel insights into the potential therapeutic implications of plasma metabolites in CKD, specifically revealing a causal link between elevated homocysteine levels and IgA (18). Bidirectional MR analysis, an extension of conventional MR, assumes a pivotal role in unraveling intricate interconnections within biological systems, including feedback loops between exposure and outcome variables (19).

To thoroughly scrutinize the causal nexus between plasma metabolites and CKD, a bidirectional MR investigation was undertaken. Recognizing the protracted nature of CKD progression, our analysis encompassed multiple endpoints, including eGFR and urine albumin creatinine ratio (UACR). The application of bidirectional MR aimed to furnish a more comprehensive understanding of the intricate connections between plasma metabolites and the evolving dynamics of renal function.

## 2 Materials and methods

### 2.1 Study design

**Figure 1:**
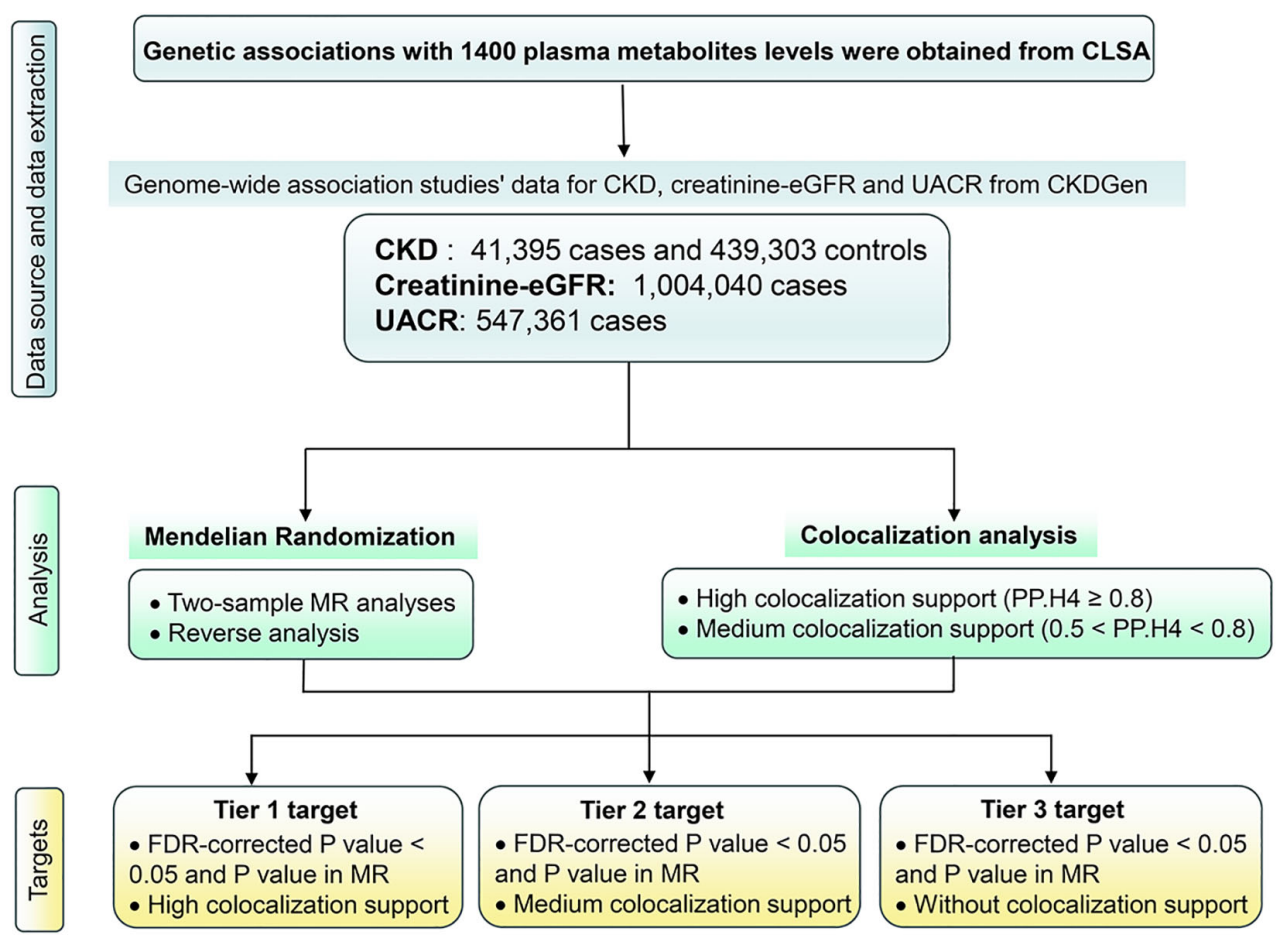
Study design. CKD, Chronic kidney disease; creatinine-eGFR, creatinine-estimated glomerular filtration rate, UACR, urine albumin creatinine ratio; FDR, false discovery rate; MR, Mendelian Randomization.

The comprehensive study design is delineated in **Figure 1**. A 2-sample MR approach was deployed, leveraging aggregated genetic associations from diverse genome-wide association studies (GWAS), to elucidate the intricate relationship between 1,400 plasma metabolites and key renal function indices, namely CKD, creatinine-eGFR, and UACR. The investigative strategy commenced with a forward MR analysis probing the connections between genetically determined plasma metabolome and renal function. Subsequently, acknowledging the potential impact of abnormal renal function on plasma metabolome levels, a reverse MR analysis was conducted to scrutinize the associations between genetically determined renal function and plasma metabolome levels. Upon establishing MR evidence of a causal effect, a colocalization analysis was executed to validate the concordance of the exposure and outcome, confirming the shared influence of the same causal variant.

### 2.2 Data source of plasma metabolites

GWAS data pertaining to 1,091 metabolites and 309 metabolite ratios were procured from 8,299 unrelated individuals of European descent participating in the Canadian Longitudinal Study on Aging (CLSA). These individuals underwent genome-wide genotyping, and their circulating plasma metabolites were carefully measured (20). Utilizing the state-of-the-art Ultrahigh Performance Liquid Chromatography-Tandem Mass Spectrometry platform, specifically the Metabolon HD4 platform by Metabolon, Inc. (Durham, NC, USA), 1,458 distinct metabolites were assayed in plasma samples. The dataset was refined by exclusively selecting metabolites with missing measurements in less than 50% of the samples, resulting in a curated set of 1,091 metabolites. Furthermore, leveraging batch-normalized metabolite levels generated by Metabolon, the dataset was further tailored to encompass metabolites with minimal missing measurements. To elucidate interrelations between metabolites, 309 pairs exhibiting shared enzymatic or transporter elements were identified via the Human Metabolome Database. Metabolite ratios within each pair were computed by dividing the batch-normalized measurement value of one metabolite by that of the other in the same individual. Subsequently, these metabolite ratios underwent careful curation, retaining only those within 3 standard deviations, followed by an inverse-rank normal transformation.

### 2.3 Data source of CKD and renal function

For instrumental variables associated with CKD and creatinine-eGFR, data were sourced from the CKDGen Consortium, constituting the primary outcomes of interest. CKD, defined as eGFR < 60 ml/min/1.73m², was discerned through GWAS data acquired from a meta-analysis involving 23 cohorts of European origin, encompassing 41,395 patients and 439,303 controls (21). Values of creatinine-eGFR were calculated from serum creatinine, using the equation for the Modification of Diet in Renal Disease (MDRD) Study and applied to define CKD (eGFRcrea <60 mL/min/1.73 m^2^) (22). Creatinine-eGFR was age and sex adjusted using residuals, and then natural log transformed (22). Creatinine-eGFR GWAS data emanated from meta-analyses conducted in the UK Biobank (n = 436,581, European origin) and the CKDGen consortium (n = 765,348, predominantly European origin) (23). In the UK Biobank, serum creatinine measurements were ascertained using a Beckman Coulter AU5800 analysis and were incorporated into the CKD Epidemiology Collaboration formula for eGFR calculation (24, 25). For individuals aged less than 18 years, the Schwartz formula was used instead (26). UACR was calculated as urinary albumin/urinary creatinine (mg/g) to account for differences in urine concentration (27). The data of UACR were derived from the CKDGen Consortium, which recorded the summary data of European-ancestry (n = 547,361) (28).

### 2.4 Filtering of single nucleotide polymorphisms

The judicious selection of appropriate single nucleotide polymorphisms (SNPs) is paramount for the effective execution of MR analysis. The foundational assumption of MR necessitates that all SNPs independently and significantly predict exposure at the genome-wide level of significance. In the forward MR analysis, we employed 22,043 SNPs associated with plasma metabolomes as instrumental variables for exposure. However, adherence to a stringent threshold of 5×10^-8^ would have resulted in the exclusion of a substantial proportion of SNPs. Consequently, we adopted a relatively permissive yet statistically significant threshold of 5×10^-6^, informed by previous research (29), to encompass the majority of SNPs associated with plasma metabolomes, imposing constraints of r^2^ < 0.001 and kb = 10,000 to mitigate potential linkage disequilibrium (LD) In the reverse MR analysis, concerning CKD and phenotypes associated with renal function, a threshold of 5×10^-8^ was applied to all instrumental variables, maintaining r^2^ < 0.001 and kb = 10,000 to address linkage disequilibrium concerns. Palindromic SNPs were scrutinized for definitive effect allele alignment, with ambiguous cases being excluded. Furthermore, SNPs weakly validated with F values (F = r^2^ × (N − 2)/(1 − r^2^) below 10 were removed to ensure the robustness of the association between instrumental variables and exposure. These rigorous screening criteria safeguard the validity of the findings in our study.

### 2.5 Two-sample MR

**Figure 2:**
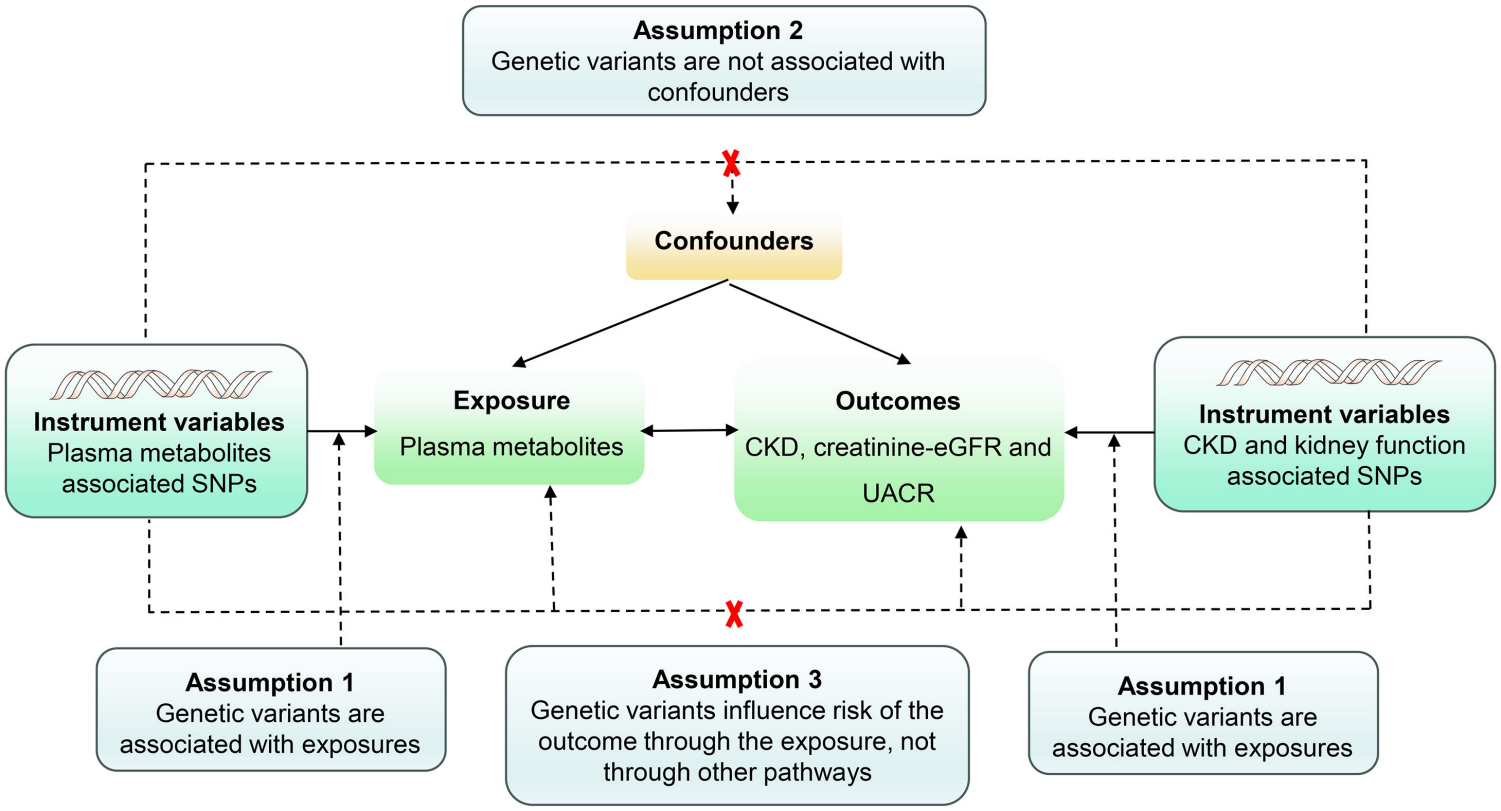
Assumptions of a mendelian randomization analysis for plasma metabolites and risk of CKD, Creatinine-eGFR and UACR. Dashed lines depict possible pleiotropic or direct causal effects between variables that would violate the assumptions of Mendelian randomization.

A detailed two-sample MR analysis was executed to assess the causal associations between plasma metabolites and both CKD and renal function (**Figure 2**). Instrumental estimates for individual SNPs were obtained using instrumental variable ratios. Assuming the validity of instruments without pleiotropy, we conducted a suite of analyses including Inverse variance weighted (IVW), MR-Egger, weighted median, weighted mode, and simple mode to scrutinize the causal relationship between exposure factors and outcomes (30, 31). Additionally, an exhaustive sensitivity analysis was performed employing diverse methodologies to ensure result reliability. This encompassed the examination of heterogeneity, assessment of horizontal pleiotropy, analysis of funnel plots, and implementation of a leave-one-out analysis. The primary MR analysis, IVW, was selected for those with two or more SNPs to evaluate the potential causative effect of plasma metabolites on the risk of CKD, creatinine-eGFR, and UACR. For plasma metabolites with only one SNP, the Wald ratio was chosen as the primary MR analysis, facilitating the evaluation of the efficacy and credibility of each instrument employed in our instrumental variable analysis. Heterogeneity in individual causal effects was assessed through Q-statistics, where p-values below 0.05 indicated the presence of heterogeneity (32). To address horizontal pleiotropy, MR Egger regression and MR pleiotropy residual sum and outlier (MR-PRESSO) test were employed for corrective purposes. Given the inherent risk of false positive outcomes arising from the simultaneous processing and comparison of multiple datasets, the Bonferroni correction test was employed to evaluate the robustness of the causal relationship between exposure and outcome variables. A two-sided P value below 3.57 × 10^-5^ (0.05/1,400) was considered significant, with estimates falling between 0.05 and 3.57 × 10^-5^ interpreted as suggestive evidence of an association in this study, considering the conservative nature of the Bonferroni correction method.

### 2.6 Colocalization

Colocalization analysis, a critical component of our investigation, was executed using the coloc R package (33) to ascertain the influence of linkage disequilibrium on the identified associations between plasma metabolites and CKD, along with renal function. Employing a Bayesian approach, the analysis contemplated five mutually exclusive hypotheses for each locus (1): no association with either trait (2); association solely with trait 1 (3); association solely with trait 2 (4); both traits being associated with distinct causal variants; and (5) both traits sharing the same causal variant. Posterior probabilities were assigned to each hypothesis test (H0, H1, H2, H3, and H4). Notably, prior probabilities for the SNP’s association with trait 1 only (p1) and trait 2 only (p2) were set at 1 × 10^-4^ each, with the probability of the SNP being associated with both traits (p3) set at 1 × 10^-5^. The robustness of colocalization evidence was delineated by posterior probabilities, with a value of PP.H4 ≥ 0.8 indicative of strong colocalization evidence, and medium colocalization indication denoted by 0.5 < PP.H4 < 0.8.

Plasma metabolites-outcome associations with FDR-corrected P value < 0.05 in MR were subsequently classified into three groups. Plasma metabolites demonstrating high-support evidence of colocalization (PP.H4 ≥ 0.8) were designated as tier 1 targets. Those with medium-support evidence of colocalization (0.5 < PP.H4 < 0.8) were classified as tier 2 targets. The remaining proteins, lacking substantial colocalization evidence, were designated as tier 3 targets. This careful classification facilitates a nuanced understanding of the colocalization strength and guides subsequent prioritization of plasma metabolites for further investigation.

## 3 Results

### 3.1 The selection of instrumental variables

A total of 1,400 plasma metabolites were included in subsequent MR analyses. We performed the quality control of SNPs. SNPs associated with plasma metabolites passed the locus-wide significance threshold of P < 5×10^−6^ was screened. Subsequently, harmonization and clumping procedures were applied, leading to the removal of palindrome SNPs and a reduction in the impact of LD. Consequently, the number of IVs pertaining to the 1,400 plasma metabolites for CKD was determined to be 7,661. Similarly, the number of IVs for the 1,400 plasma metabolites associated with creatinine-eGFR was 7,260, while for UACR, it was 7,122. Notably, all F-statistics of IVs surpassed the threshold of 10, signifying the absence of weak instrument bias (**Supplementary Tables S1**-**S3**).

### 3.2 Causal link between plasma metabolites and CKD

**Figure 3:**
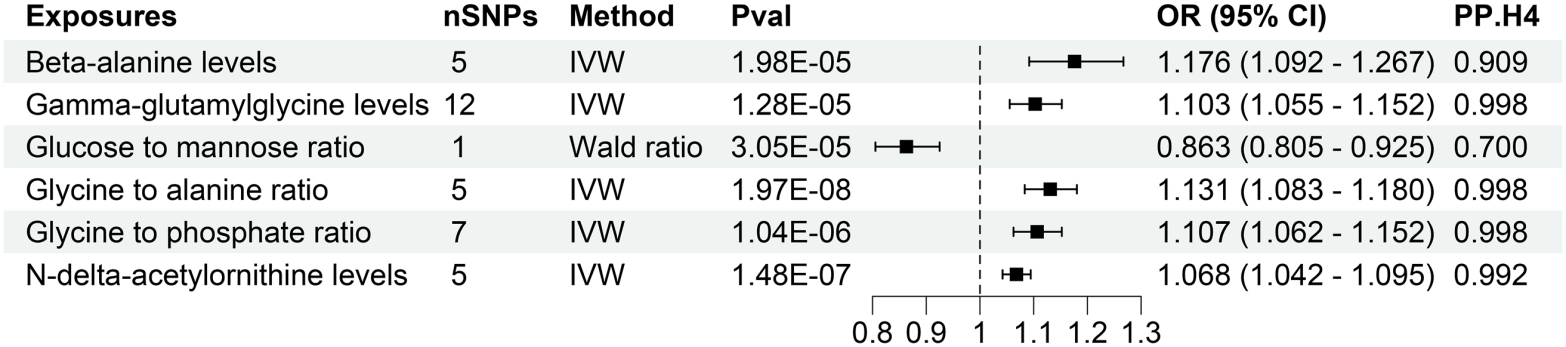
Forest of plasma metabolites with risk of chronic kidney disease. nSNPs, number of single nucleotide polymorphisms; Pval, P-value; OR, odds ratio; CI, confidence interval; PP.H4, posterior probability of hypothesis 4.

The investigation substantiates a causative link between 6 plasma metabolites and an elevated susceptibility to CKD (**Figure 3**, **Supplementary Table S4**). Employing the IVW method for genetic prediction, higher levels of Beta-alanine, Gamma-glutamylglycine, Glycine to alanine ratio, Glycine to phosphate ratio, and N-delta-acetylornithine were associated with an augmented risk of CKD, as evidenced by odds ratios (OR) and 95% confidence intervals (CI). Specifically, Beta-alanine levels (OR = 1.176, 95% CI 1.092-1.267, P = 1.98×10^-5^), Gamma-glutamylglycine levels (OR = 1.103, 95% CI 1.055-1.152, P = 1.28×10^-5^), Glycine to alanine ratio (OR = 1.131, 95% CI 1.083-1.180, P = 1.97×10^-8^), Glycine to phosphate ratio (OR = 1.107, 95% CI 1.062-1.152, P = 1.04×10^-6^), and N-delta-acetylornithine levels (OR = 1.068, 95% CI 1.042-1.095, P = 1.48×10^-7^) exhibited a consistent association with an increased CKD risk, as corroborated by MR-Egger and weighted median analyses.

Contrastingly, Wald analysis demonstrated that an increase in the Glucose to mannose ratio was associated with a decreased risk of CKD (OR = 0.863, 95% CI 0.805-0.925, P = 3.05×10^-5^). Further assessments, including MR-Egger regression, MR-PRESSO and Cochrane’s Q test, substantiated the absence of horizontal pleiotropy, outliers, or notable heterogeneity in the selected SNPs (P > 0.05). Comprehensive presentations of individual SNP effects and the cumulative effects of each analytical method are provided in **Supplementary Figures S1**, **S2**. Additionally, the robustness of the estimated causal effect was reaffirmed through a “leave-one-out” analysis, demonstrating no significant alteration when each SNP was individually excluded (**Supplementary Figure S3**).

### 3.3 Causal link between plasma metabolites and creatinine-eGFR

**Figure 4:**
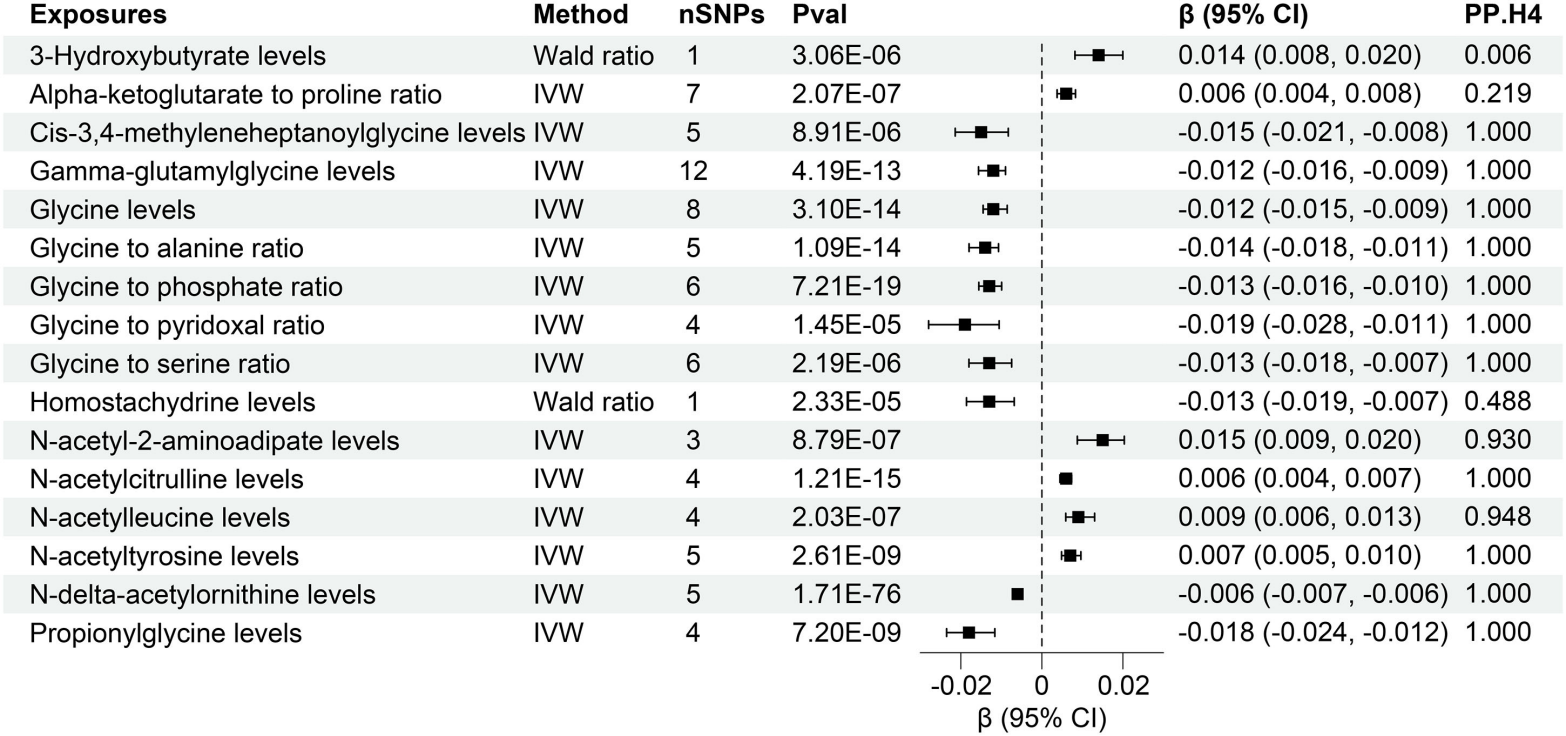
Forest of plasma metabolites with risk of estimated glomerular filtration rate. nSNPs, number of single nucleotide polymorphisms; Pval, P-value; CI, confidence interval; PP.H4, posterior probability of hypothesis 4.

The research discovered evidence of a cause-and-effect relationship between 16 plasma metabolites and altered levels of creatinine-eGFR. (**Figure 4**, **Supplementary Table S5**). The IVW method for genetic prediction revealed that elevated levels of Alpha-ketoglutarate to proline ratio (β = 0.006, 95% CI (0.004, 0.008), P = 2.07×10^-7^), N-acetyl-2-aminoadipate levels (β = 0.015, 95% CI (0.007, 0.009), P = 8.79×10^-7^), N-acetylcitrulline levels (β = 0.006, 95% CI (0.004, 0.007), P = 1.21×10^-15^), N-acetylleucine levels (β = 0.009, 95% CI (0.006, 0.0013), P = 2.03×10^-7^),N-acetyltyrosine levels (β = 0.007, 95% CI (0.005, 0.010), P = 2.61×10^-9^) were associated with increased creatinine-eGFR, and the results were similar with the MR-Egger and weighted median analyses. The Wald analysis revealed that higher levels of 3-Hydroxybutyrate levels (β = 0.014, 95% CI (0.008, 0.020), P = 3.06×10^-6^) were associated with increased creatinine-eGFR.

However, the IVW analysis revealed that higher levels of Cis-3,4-methyleneheptanoylglycine levels (β = -0.015, 95% CI (-0.021, -0.008), P = 8.91×10^-6^), Gamma-glutamylglycine levels (β = -0.012, 95% CI (-0.016, -0.009), P = 4.19×10^-13^), Glycine levels (β = -0.012, 95% CI (-0.015, -0.009), P = 3.10×10^-14^), Glycine to alanine ratio (β = -0.014, 95% CI (-0.018, -0.011), P = 1.09×10^-14^), Glycine to phosphate ratio (β = -0.013, 95% CI (-0.016, -0.010), P = 7.21×10^-19^), Glycine to pyridoxal ratio (β = -0.019, 95% CI (-0.028, -0.011), P = 1.45×10^-5^), Glycine to serine ratio (β = -0.013, 95% CI (-0.018- -0.007), P = 2.19×10^-6^), N-delta-acetylornithine levels (β = -0.006, 95% CI (-0.007, -0.006), P = 1.71×10^-76^), Propionylglycine levels (β = -0.018, 95% CI (-0.024, -0.012), P = 7.20×10^-9^) were associated with decreased creatinine-eGFR. The Wald analysis revealed that that lower levels of Homostachydrine levels (β = -0.013, 95% CI (-0.019, -0.007), P = 2.33×10^-5^) were associated with increased creatinine-eGFR.

Notably, MR-Egger regression and MR-PRESSO demonstrated the absence of horizontal pleiotropy or outliers (P > 0.05), and Cochrane’s Q test results indicated no apparent heterogeneity in the selected SNPs (P > 0.05). **Supplementary Figures S4**, **S5** illustrates the effects of each SNP locus on creatinine-eGFR, emphasizing the consistency of findings across various analytical methods. Furthermore, a “leave-one-out” analysis demonstrated no significant alteration in the estimated causal effect when individual SNPs were systematically excluded, confirming the robustness of the study’s findings (**Supplementary Figure S6**).

### 3.4 Causal link between plasma metabolites and UACR

**Figure 5:**
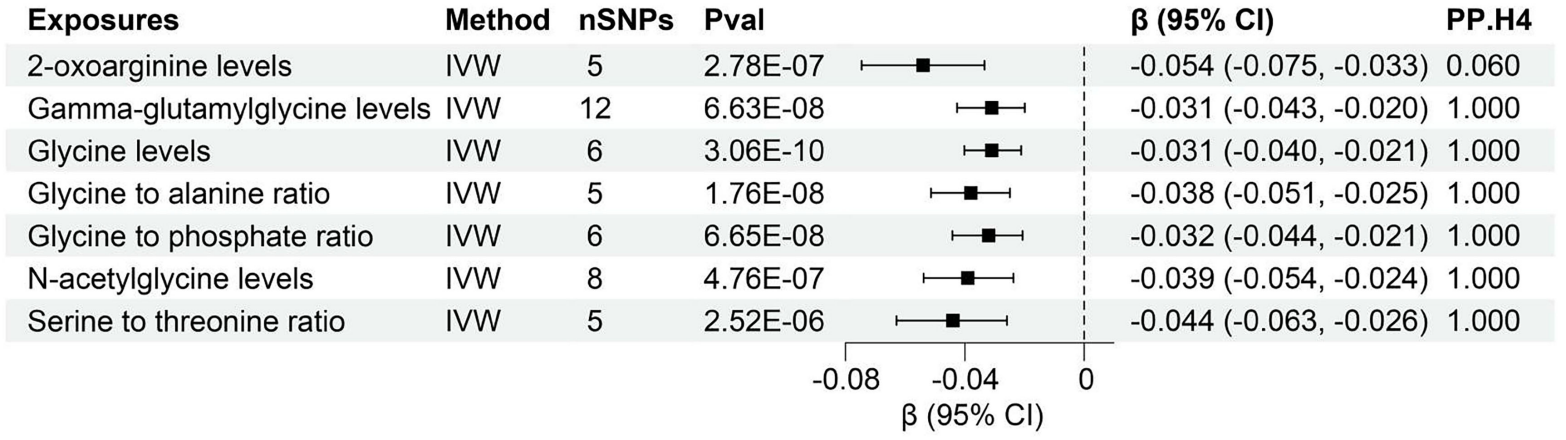
Forest of plasma metabolites with risk of urine albumin creatinine ratio. nSNPs, number of single nucleotide polymorphisms; Pval, P-value; CI, confidence interval; PP.H4, posterior probability of hypothesis 4.

The study found evidence of a causal link between 7 plasma metabolites and an increased risk of declined UACR (**Figure 5**, **Supplementary Table S6**). The IVW method for genetic prediction revealed that higher levels of 2-oxoarginine levels (β = -0.054, 95% CI (-0.075, -0.033), P = 2.78×10^-7^), Gamma-glutamylglycine levels (β = -0.031, 95% CI (-0.043, -0.020), P = 6.63×10^-8^), Glycine levels (β = -0.031, 95% CI (-0.040, -0.021), P = 3.06×10^-10^), Glycine to alanine ratio (β = -0.038, 95% CI (-0.051, -0.025), P = 1.76×10^-8^), Glycine to phosphate ratio (β = -0.032, 95% CI (-0.044, -0.021), P = 6.65×10^-8^), N-acetylglycine levels (β = -0.039, 95% CI (-0.054, -0.024), P = 4.76×10^-7^), Serine to threonine ratio (β = -0.044, 95% CI (-0.063, -0.026), P = 2.52×10^-6^) were associated with decreased UACR.

Notably, the MR-Egger regression and MR-PRESSO demonstrated an absence of horizontal pleiotropy or outliers (P > 0.05). While Q-statistics indicated some evidence of heterogeneity in the analysis of Gamma-glutamylglycine levels, Glycine to phosphate ratio, N-acetylglycine levels, Serine to threonine ratio, and Creatine levels, no such evidence was found in the analysis of other plasma metabolites. **Supplementary Figures S7**, **S8** displays the individual effects of each SNP and the combined effects of each analytical method, emphasizing the robustness of the findings across various approaches. Moreover, a “leave-one-out” analysis demonstrated no significant alteration in the estimated causal effect when individual SNPs were systematically excluded, reaffirming the study’s findings (**Supplementary Figure S9**).

### 3.5 Colocalization

We conducted rigorous colocalization analyses investigating the potential shared causal variants between plasma metabolites and CKD as well as renal function. This investigation aimed to determine whether the identified associations of plasma metabolites with CKD and renal function emanated from the same underlying genetic factors (**Supplementary Figures S10**-**S12**). The results revealed robust evidence of high-support colocalization between 5 plasma metabolites, namely Beta-alanine levels, Gamma-glutamylglycine levels, Glycine to alanine ratio, Glycine to phosphate ratio, and N-delta-acetylornithine levels, and CKD, designating them as tier 1 targets (**Figure 3**). Additionally, a noteworthy medium level of evidence for co-localization was established between Glucose-to-mannose ratio and CKD, categorizing it as tier 2 (**Figure 3**).

Furthermore, colocalization analysis uncovered high-support evidence for the association of 13 plasma metabolites, including Cis-3,4-methyleneheptanoylglycine levels, Gamma-glutamylglycine levels, Glycine levels, Glycine to alanine ratio, Glycine to phosphate ratio, Glycine to pyridoxal ratio, Glycine to serine ratio, N-acetyl-2-aminoadipate levels, N-acetylcitrulline levels, N-acetylleucine levels, N-acetyltyrosine levels, N-delta-acetylornithine levels, Propionylglycine levels, and with renal function (creatinine-eGFR). These metabolites were identified as tier 1 targets, indicating a robust and well-supported relationship (**Figure 4**). Moreover, a similar high level of colocalization evidence was observed between 6 plasma metabolites, namely Gamma-glutamylglycine levels, Glycine levels, Glycine to alanine ratio, Glycine to phosphate ratio, N-acetylglycine levels, Serine to threonine ratio and UACR, designating them as tier 1 targets (**Figure 5**). Those plasma metabolite-outcome pairs with limited evidence of colocalization were appropriately classified as tier 3 targets.

### 3.6 Reverse analysis

**Figure 6:**
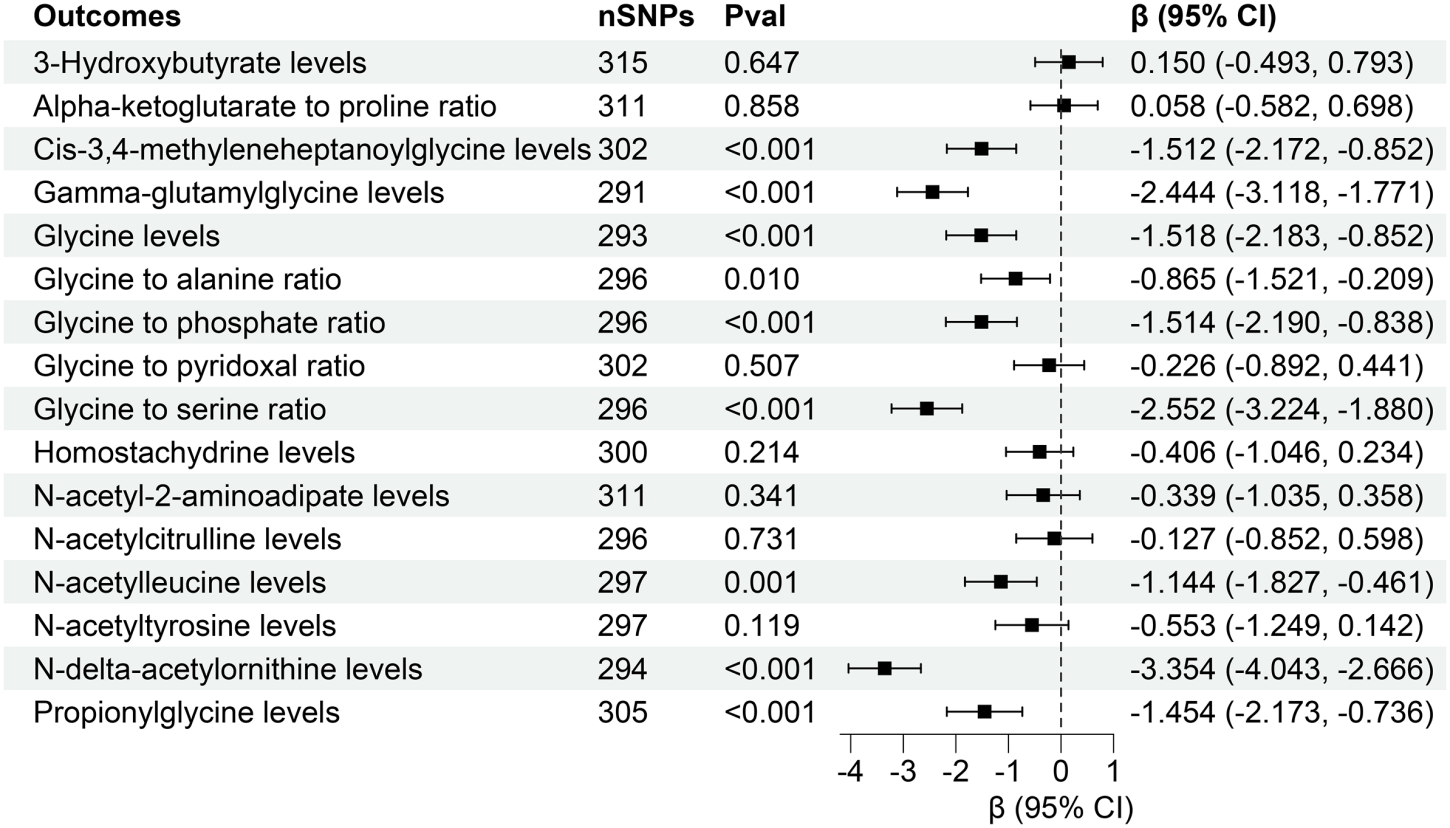
Forest for association of genetically predicted estimated glomerular filtration rate with plasma metabolites. nSNPs, number of single nucleotide polymorphisms; Pval, P-value; CI, confidence interval.

This investigation revealed compelling causal associations between 21 plasma metabolites and diverse CKD outcomes through forward MR analyses. In a complementary reverse MR analysis, we explored the genetic relationship between CKD and these 21 plasma metabolites. The IVW analysis unveiled that higher levels of Cis-3,4-methyleneheptanoylglycine levels (β = -1.512, 95% CI (-2.172, -0.852), P < 0.001), Gamma-glutamylglycine levels (β = -2.444, 95% CI (-3.118, -1.771), P < 0.001), Glycine levels (β = -1.518, 95% CI (-2.183, -0.852), P < 0.001), Glycine to alanine ratio (β = -0.865, 95% CI (-1.521, -0.209), P = 0.010), Glycine to phosphate ratio (β = -1.514, 95% CI (-2.190, -0.838), P < 0.001), Glycine to serine ratio (β = -2.552, 95% CI (-3.224, -1.880), P < 0.001), N-acetylleucine levels (β = -1.144, 95% CI (-1.827, -0.461), P = 0.001), N-delta-acetylornithine levels (β = -3.354, 95% CI (-4.043, -2.666), P < 0.001), Propionylglycine levels (β = -1.454 95% CI (-2.173, -0.736), P < 0.001) might contribute to lower eGFR (**Figure 6**).

**Figure 7:**
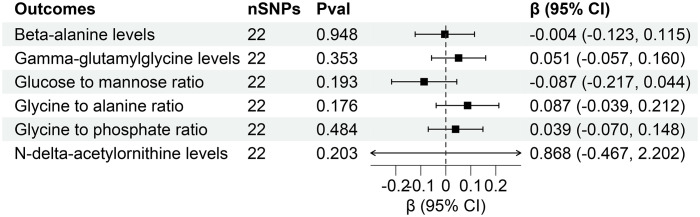
Forest for association of genetically predicted chronic kidney disease with plasma metabolites. nSNPs, number of single nucleotide polymorphisms; Pval, P-value; CI, confidence interval.

**Figure 8:**
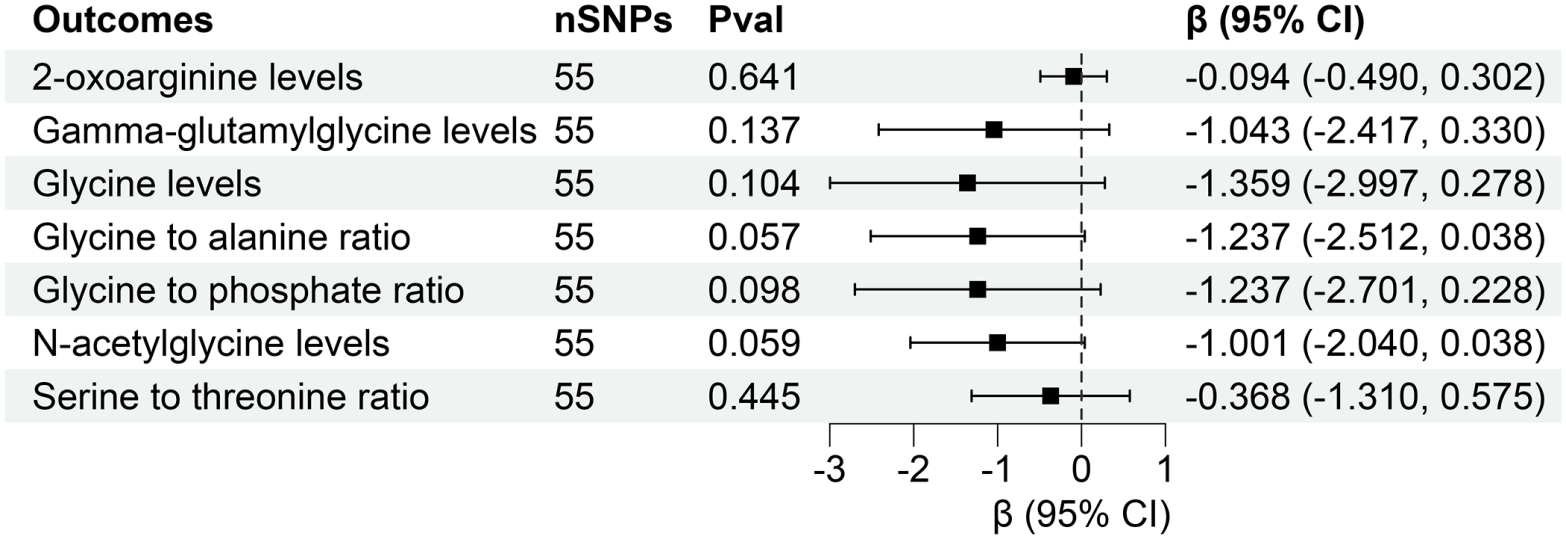
Forest for association of genetically predicted urine albumin creatinine ratio with plasma metabolites. nSNPs, number of single nucleotide polymorphisms; Pval, P-value; CI, confidence interval.

However, reverse MR analysis showed that eGFR did not affect the levels of other plasma metabolites. Similarly, CKD and UACR did not affect the levels of corresponding metabolites (**Figures 7**, **8**). Importantly, throughout the analysis, no evidence of horizontal pleiotropy was identified (P > 0.05), suggesting the robustness and reliability of our findings (**Supplementary Tables S10**-**S12**).

## 4 Discussion

Our investigation is firmly grounded in innovative and expansive GWAS data, employing a gene prediction methodology to elucidate the intricate link between plasma metabolites and the initiation and progression of CKD. As a result, our obtained findings not only carry heightened credibility but also bear a reliable interpretive impact causally, thereby potentially guiding future CKD treatments by targeting specific plasma metabolites.

In the Two-sample MR results, various plasma metabolites have been subject to extensive scrutiny in the realm of CKD. The work of Yu et al. (34), employing a combination of high-throughput metabolomic and genomic technologies, illuminated that a mutation in NAT8 is associated with N-acetylornithine, N-acetyl-1-methylhistidine, and eGFR, consequently influencing the risk of incident CKD. These findings substantiate the role of N-acetylation in CKD progression (35). Our current investigation underscores the critical and actively researched aspect of exploring the functional repercussions of the underlying causal variants. Our research echoes similar conclusions, particularly in associating elevated N-delta-acetylornithine levels with lower eGFR. Notably, both Propionylglycine and Cis-3,4-methyleneheptanoylglycine belong to acylglycines, compounds formed by the amalgamation of a fatty acid and glycine. The interaction between glycine and tyrosine to form bile salts aligns with the study by Lin et al. (36), discerning global serum profiles in CKD patients and healthy controls, emphasizing the pivotal role of the broader distribution of free fatty acids in driving CKD progression. This consistency in findings provides not only enhanced insights into CKD pathophysiological changes but also hints at potential therapeutic avenues.

Furthermore, 3-Hydroxybutyrate, an endogenously produced ketone body molecule in the liver, adds a crucial dimension to our understanding. A mounting body of evidence underscores the potential positive impact of ketone bodies in diverse diseases, including those with implications for renal health. The study involving mice subjected to 5/6 nephrectomy revealed that treatment with 1,3-butanediol effectively mitigated the progression of proteinuria, glomerular, and tubulointerstitial injury (37). This discovery holds profound clinical implications, signifying a potential avenue for intervention-supplementing exogenous ketone bodies to enhance renal function and protract the progression of CKD.

Examining serum disparities between CKD patients and healthy individuals, Chen et al. (38) elucidated that pyridoxal may elevate homocysteine levels and oxidative stress. Conversely, alanine and serine, known for their antioxidant properties (39, 40), could mitigate oxidative stress and protect the kidneys from damage. Another study highlighted phosphate’s role in regulating the synthesis and release of various hormones in the kidneys, including parathyroid hormone, vitamin D, and the renin-angiotensin-aldosterone system (41). These hormones significantly influence renal function and electrolyte balance, and phosphate aids in protecting renal function by regulating their secretion. Our research aligns with these findings, indicating that increased glycine to pyridoxal ratio, glycine to alanine ratio, glycine to phosphate ratio, or glycine to serine ratio, along with a decline in Serine to threonine ratio, impairs renal function.

Notably, N-acetylcitrulline and 2-oxoarginine, participants in the arginine synthesis pathway as metabolites of citrulline (42), have been relatively unexplored in their regulatory pathways impacting renal function. Drawing from existing research, we speculate that the conversion of arginine back to ornithine through nitric oxide synthase in the arginine-guanidino pathway, releasing nitric oxide (NO) (43), might help maintain a certain renal blood flow and filtration rate, thereby protecting renal function. This discovery paves the way for a novel research direction aimed at enhancing or delaying CKD through a targeted exploration of plasma metabolites. Our findings indicate that certain metabolites, such as 2-oxoarginine levels, Gamma-glutamylglycine levels, Glycine levels, and their respective ratios (e.g., Glycine to alanine ratio), are negatively correlated with UACR. This suggests that higher levels of these metabolites are associated with lower UACR, which is protective since a high UACR indicates albuminuria, a pathological condition. This protective association highlights the potential of these metabolites as biomarkers or therapeutic targets in CKD management.

In the Two-sample MR analyses, our findings revealed divergent roles of certain metabolites in CKD, contrary to established studies. Ikeda’s investigation into the impact of renal insufficiency on plasma amino acid levels contradicted previous assumptions, indicating no significant difference in glycine levels between the control and renal insufficiency groups (44). Our results propose a novel perspective, indicating an inverse correlation-increased plasma glycine associates with a progressive decline in eGFR. As indicated by previous research, glycine functions as a major osmolyte in the renal medulla (45). The observed abnormal changes in organic osmolyte levels, as noted in individuals with renal cell carcinoma (RCC) (36, 46), suggest a potential disruption in kidney cell osmolar function among CKD patients. Additionally, despite the documented antioxidative effects of β-Hydroxybutyrate (β-HB) via Nrf2 signaling, which mitigates kidney hypertrophy in diabetic mice, our study implicates beta-alanine in CKD occurrence, albeit without elucidating specific mechanisms—a recognized limitation (47). Nonetheless, this finding aligns with the typical circulating amino acid profile observed in individuals with kidney disease (48, 49). Finally, our research challenges conventional wisdom by asserting that an increased glucose-to-mannose ratio protects renal function, thereby delaying CKD progression. This intriguing conclusion may be linked to mannose’s established predictive ability for CKD development (50). Despite the uncertainties and limitations inherent in our study, this novel insight contributes to the evolving understanding of the intricate relationships between metabolites and CKD, paving the way for future investigations into disease mechanisms and potential therapeutic interventions.

In our extended colocalization analysis, robust evidence of colocalization emerged for 16 plasma metabolites with CKD and renal function, designating them as tier 1. Of particular emphasis is a potential causal variant, rs1047891, identified proximal to CPS1, encoding the mitochondrial enzyme carbamoyl-phosphate synthase 1. This enzyme governs the synthesis of carbamoyl phosphate from ammonia, playing a pivotal role in the urea cycle. Noteworthy clinical manifestations, including hyperammonemia, are evident in individuals with CPS1 deficiency (51, 52). Thus, rs1047891 may potentially influence CKD development by modulating CPS1 gene expression. This discovery bolsters the outcomes of MR analyses and holds crucial implications for gene editing interventions to control CKD occurrence in the future.

Our research significantly advances the understanding of the bidirectional relationship between plasma metabolites and CKD. The response of plasma metabolites acts not only as a trigger for CKD but also as a consequence of renal dysfunction. While prior studies predominantly focused on factors leading to CKD development, neglecting the reciprocal impact of renal disease on plasma metabolites (53–55), our findings illuminate that renal impairment induces alterations in serum metabolites through diverse pathways. A primary pathway involves the kidneys’ compromised ability to filter metabolites, leading to their accumulation in the bloodstream (56). Changes in renal metabolic pathways affect the generation and clearance of metabolites, exacerbating the endocrine metabolic regulation imbalance prompted by renal impairment and influencing serum metabolite levels (5, 57). Our research also found that n-delta-acetylornithine levels, cis-3,4-methyleneheptanoylglycine levels, gamma-glutamylglycine levels and glycine to serine ratio could be changed with the development of CKD. This finding contributes to our better understanding of the relationship between renal function and plasma metabolites, providing new directions for future research on inhibiting the progression of CKD.

Our findings reveal complex associations between plasma metabolites, UACR, CKD, and eGFR. It is important to clarify that while a decrease in UACR is generally considered protective because high UACR is indicative of kidney damage, the metabolites that are negatively correlated with UACR (e.g., Glycine to alanine ratio) might also be associated with increased CKD risk and decreased eGFR. This apparent contradiction can be attributed to the multifaceted role of these metabolites in kidney function and disease progression. For example, while lower UACR might suggest reduced albuminuria and potential protection, other metabolic changes could be influencing the overall risk of CKD and eGFR decline.

This MR study, leveraging recent pooled data, marks the inaugural exploration into the causal interplay between CKD and plasma metabolites. Departing from conventional observational studies susceptible to reverse causality bias involving non-renal metabolic pathways, our MR analysis mitigates confounding factors, offering a dependable estimate of causality. Notably, our study not only incorporates a dedicated GWAS cohort for CKD events but also integrates diverse dynamic indicators of renal function and phenotypes, rendering the association between renal disease and plasma metabolites more clinically relevant and informative. The accessibility of serum, a readily obtainable biological specimen in clinical practice, underscores the substantial implications of our findings for future clinical research in relevant domains.

Nevertheless, several limitations warrant acknowledgment. Firstly, the genetic data predominantly stem from individuals of European descent, necessitating caution in extending the findings to other ethnic groups. Despite diligent efforts to exclude SNPs linked to potential confounders and perform sensitivity analyses, there remains a possibility of undetected complex and multidirectional effects. The use of instrumental variables from the GWAS meta-analysis precludes exploration of potential stratification effects and nonlinear relationships, necessitating further investigation. While stringent correction thresholds and pleiotropy checks were implemented to ensure robust MR results, this approach may lead to some false-negative results. Moreover, MR analysis, while robust for estimating causality, should not substitute for RCTs. Consequently, the causality inferred from this study might not perfectly align with RCT observations, emphasizing the need for individual-based genetic observations and potential integration of RCTs in future research to validate identified causal relationships. Finally, Creatinine, a byproduct of muscle metabolism, indeed has limitations that could potentially introduce bias, particularly in populations with varying muscle mass. As decreased muscle mass can lead to lower serum creatinine levels, which may falsely suggest higher eGFR values, potentially masking renal impairment. Recent studies have shown that some SNPs and associated metabolites correlate with decreased muscle mass. For instance, Fujimoto and Jin found he alpha-actinin-3 rs1815739 genotype (58), RPS10, NUDT3, and GPD1L (59) are correlated with decreased muscle mass through their respective studies. Similarly, other researchers also found CYP2R1 (rs10741657), GC (rs2282679), VDR (rs10741657) (60) and Cav1 G14713A (61) could lead to a loss of muscle mass. Furthermore, Some metabolites, such as adenine nucleotides, NAD (62), serum let-7e-5p (63), and butyrate (64), can also contribute to the loss of muscle mass. However, in the conclusions derived from this study, no related literature was found indicating that the metabolites causally associated with increased creatinine-eGFR might lead to decreased muscle mass. This suggests that our conclusions have a certain level of credibility and persuasiveness. Future research should incorporate alternative biomarkers of kidney function that are less influenced by muscle mass, such as cystatin C, which may provide a more accurate assessment of renal function in diverse populations.

## 5 Conclusion

In summary, through an extensive genome-wide association study and bidirectional MR analyses, we unveil associations between 21 metabolites and CKD, creatinine-eGFR, and UACR. Causal links established highlight potential therapeutic targets. They are respectively 3-Hydroxybutyrate levels, Alpha-ketoglutarate to proline ratio, Cis-3,4-methyleneheptanoylglycine levels, Gamma-glutamylglycine levels, Glycine levels, Glycine to alanine ratio, Glycine to phosphate ratio, Glycine to pyridoxal ratio, Glycine to serine ratio, Homostachydrine levels, N-acetyl-2-aminoadipate levels, N-acetylcitrulline levels, N-acetylleucine levels, N-acetyltyrosine levels, N-delta-acetylornithine levels, Propionylglycine levels, Beta-alanine levels, Glucose to mannose ratio, 2-oxoarginine levels, N-acetylglycine levels, Serine to threonine ratio. Colocalization analysis strengthens these findings, demonstrating robust evidence of shared genetic variants for 16 plasma metabolites with CKD, eGFR, and UACR, all classified as tier 1. These outcomes deepen our comprehension of metabolite-driven mechanisms in CKD progression, opening avenues for targeted interventions and personalized treatment strategies to alleviate the burden of this prevalent disease.

## Data availability statement

The original contributions presented in the study are included in the article/**Supplementary Material**. Further inquiries can be directed to the corresponding authors.

## Author contributions

XZ: Writing – original draft, Writing – review & editing. KK: Conceptualization, Data curation, Writing – review & editing. HZ: Funding acquisition, Resources, Writing – review & editing. XW: Data curation, Formal analysis, Writing – review & editing. XG: Supervision, Validation, Writing – review & editing. YW: Supervision, Validation, Writing – review & editing. JG: Conceptualization, Investigation, Software, Writing – review & editing. FL: Investigation, Software, Supervision, Writing – review & editing.

## References

[1] Eckardt KU Coresh J Devuyst O Johnson RJ Köttgen A Levey AS. Evolving importance of kidney disease: from subspecialty to global health burden. Lancet. (2013) 382:158–69. doi: 10.1016/S0140-6736(13)60439-0 23727165

[2] Bowden J Davey Smith G Haycock PC Burgess S. Consistent estimation in mendelian randomization with some invalid instruments using a weighted median estimator. Genet Epidemiol. (2016) 40:304–14. doi: 10.1002/gepi.21965 PMC4849733 27061298

[3] Kalantar-Zadeh K Jafar TH Nitsch D Neuen BL Perkovic V. Chronic kidney disease. Lancet. (2021) 398:786–802. doi: 10.1016/S0140-6736(21)00519-5 34175022

[4] Baek J He C Afshinnia F Michailidis G Pennathur S. Lipidomic approaches to dissect dysregulated lipid metabolism in kidney disease. Nat Rev Nephrol. (2022) 18:38–55. doi: 10.1038/s41581-021-00488-2 34616096 PMC9146017

[5] Mitrofanova A Merscher S Fornoni A. Kidney lipid dysmetabolism and lipid droplet accumulation in chronic kidney disease. Nat Rev Nephrol. (2023) 19:629–45. doi: 10.1038/s41581-023-00741-w PMC12926870 37500941

[6] Yoshioka K Hirakawa Y Kurano M Ube Y Ono Y Kojima K. Lysophosphatidylcholine mediates fast decline in kidney function in diabetic kidney disease. Kidney Int. (2022) 101:510–26. doi: 10.1016/j.kint.2021.10.039 34856312

[7] Yang T Richards EM Pepine CJ Raizada MK. The gut microbiota and the brain-gut-kidney axis in hypertension and chronic kidney disease. Nat Rev Nephrol. (2018) 14:442–56. doi: 10.1038/s41581-018-0018-2 PMC6385605 29760448

[8] Tuttle KR Bakris GL Bilous RW Chiang JL de Boer IH Goldstein-Fuchs J. Diabetic kidney disease: a report from an ADA Consensus Conference. Am J Kidney Dis. (2014) 64:510–33. doi: 10.1053/j.ajkd.2014.08.001 25257325

[9] Herman-Edelstein M Scherzer P Tobar A Levi M Gafter U. Altered renal lipid metabolism and renal lipid accumulation in human diabetic nephropathy. J Lipid Res. (2014) 55:561–72. doi: 10.1194/jlr.P040501 PMC3934740 24371263

[10] Breyer MD Susztak K. The next generation of therapeutics for chronic kidney disease. Nat Rev Drug Discovery. (2016) 15:568–88. doi: 10.1038/nrd.2016.67 PMC5511522 27230798

[11] Ritter CS Slatopolsky E. Phosphate toxicity in CKD: the killer among us. Clin J Am Soc Nephrol. (2016) 11:1088–100. doi: 10.2215/CJN.11901115 PMC4891758 26912542

[12] Chen DQ Cao G Chen H Liu D Su W Yu XY. Gene and protein expressions and metabolomics exhibit activated redox signaling and wnt/β-catenin pathway are associated with metabolite dysfunction in patients with chronic kidney disease. Redox Biol. (2017) 12:505–21. doi: 10.1016/j.redox.2017.03.017 PMC5369369 28343144

[13] Tripepi G Kollerits B Leonardis D Yilmaz MI Postorino M Fliser D. Competitive interaction between fibroblast growth factor 23 and asymmetric dimethylarginine in patients with CKD. J Am Soc Nephrol. (2015) 26:935–44. doi: 10.1681/ASN.2013121355 PMC4378102 25150156

[14] Li DY Tang WHW. Contributory role of gut microbiota and their metabolites toward cardiovascular complications in chronic kidney disease. Semin Nephrol. (2018) 38:193–205. doi: 10.1016/j.semnephrol.2018.01.008 29602401 PMC5881581

[15] Lawlor DA Harbord RM Sterne JA Timpson N Davey Smith G. Mendelian randomization: using genes as instruments for making causal inferences in epidemiology. Stat Med. (2008) 27:1133–63. doi: 10.1002/sim.3034 17886233

[16] Fall T Hägg S Mägi R Ploner A Fischer K Horikoshi M. The role of adiposity in cardiometabolic traits: a Mendelian randomization analysis. PloS Med. (2013) 10:e1001474. doi: 10.1371/journal.pmed.1001474 23824655 PMC3692470

[17] Holmes MV Lange LA Palmer T Lanktree MB North KE Almoguera B. Causal effects of body mass index on cardiometabolic traits and events: a Mendelian randomization analysis. Am J Hum Genet. (2014) 94:198–208. doi: 10.1016/j.ajhg.2013.12.014 24462370 PMC3928659

[18] Zhang YM Zhou XJ Shi SF Liu LJ Lyu JC Zhang H. Homocysteine and IgA nephropathy: observational and Mendelian randomization analyses. Chin Med J (Engl). (2020) 133:277–84. doi: 10.1097/CM9.0000000000000613 PMC7004620 31929371

[19] Richmond RC Davey Smith G Ness AR den Hoed M McMahon G Timpson NJ. Assessing causality in the association between child adiposity and physical activity levels: a Mendelian randomization analysis. PloS Med. (2014) 11:e1001618. doi: 10.1371/journal.pmed.1001618 24642734 PMC3958348

[20] Chen Y Lu T Pettersson-Kymmer U Stewart ID Butler-Laporte G Nakanishi T. Genomic atlas of the plasma metabolome prioritizes metabolites implicated in human diseases. Nat Genet. (2023) 55:44–53. doi: 10.1038/s41588-022-01270-1 36635386 PMC7614162

[21] Wuttke M Li Y Li M Sieber KB Feitosa MF Gorski M. A catalog of genetic loci associated with kidney function from analyses of a million individuals. Nat Genet. (2019) 51:957–72. doi: 10.1038/s41588-019-0407-x PMC6698888 31152163

[22] Pattaro C Teumer A Gorski M Chu AY Li M Mijatovic V. Genetic associations at 53 loci highlight cell types and biological pathways relevant for kidney function. Nat Commun. (2016) 7:10023. doi: 10.1038/ncomms10023 26831199 PMC4735748

[23] Stanzick KJ Li Y Schlosser P Gorski M Wuttke M Thomas LF. Discovery and prioritization of variants and genes for kidney function in >1. 2 million individuals. Nat Commun. (2021) 12:4350. doi: 10.1038/s41467-021-24491-0 34272381 PMC8285412

[24] Pattaro C Riegler P Stifter G Modenese M Minelli C Pramstaller PP. Estimating the glomerular filtration rate in the general population using different equations: effects on classification and association. Nephron Clin Pract. (2013) 123:102–11. doi: 10.1159/000351043 23797027

[25] Inker LA Schmid CH Tighiouart H Eckfeldt JH Feldman HI Greene T. Estimating glomerular filtration rate from serum creatinine and cystatin C. N Engl J Med. (2012) 367:20–9. doi: 10.1056/NEJMoa1114248 PMC4398023 22762315

[26] Schwartz GJ Schneider MF Maier PS Moxey-Mims M Dharnidharka VR Warady BA. Improved equations estimating GFR in children with chronic kidney disease using an immunonephelometric determination of cystatin C. Kidney Int. (2012) 82:445–53. doi: 10.1038/ki.2012.169 PMC3433576 22622496

[27] Teumer A Tin A Sorice R Gorski M Yeo NC Chu AY. Genome-wide association studies identify genetic loci associated with albuminuria in diabetes. Diabetes. (2016) 65:803–17. doi: 10.2337/db15-1313 PMC4764151 26631737

[28] Teumer A Li Y Ghasemi S Prins BP Wuttke M Hermle T. Genome-wide association meta-analyses and fine-mapping elucidate pathways influencing albuminuria. Nat Commun. (2019) 10:4130. doi: 10.1038/s41467-019-11576-0 31511532 PMC6739370

[29] Chen L Qiu W Sun X Gao M Zhao Y Li M. Novel insights into causal effects of serum lipids and lipid-modifying targets on cholelithiasis. Gut. (2024) 73(3):521–32. doi: 10.1136/gutjnl-2023-330784 37945330

[30] Tan JS Liu NN Guo TT Hu S Hua L. Genetically predicted obesity and risk of deep vein thrombosis. Thromb Res. (2021) 207:16–24. doi: 10.1016/j.thromres.2021.08.026 34507265

[31] Burgess S Butterworth A Thompson SG. Mendelian randomization analysis with multiple genetic variants using summarized data. Genet Epidemiol. (2013) 37:658–65. doi: 10.1002/gepi.21758 PMC4377079 24114802

[32] Burgess S Bowden J Dudbridge F Thompson SG. Robust instrumental variable methods using multiple candidate instruments with application to Mendelian randomization. arXiv: Method. (2016) 18(8):4865–78. doi: 10.48550/arXiv.1606.03729

[33] Giambartolomei C Vukcevic D SChadt EE Franke L Hingorani AD Wallace C. Bayesian test for colocalisation between pairs of genetic association studies using summary statistics. PloS Genet. (2014) 10:e1004383. doi: 10.1371/journal.pgen.1004383 24830394 PMC4022491

[34] Yu B Zheng Y Alexander D Morrison AC Coresh J Boerwinkle E. Genetic determinants influencing human serum metabolome among African Americans. PloS Genet. (2014) 10:e1004212. doi: 10.1371/journal.pgen.1004212 24625756 PMC3952826

[35] Suhre K Shin SY Petersen AK Mohney RP Meredith D Wägele B. Human metabolic individuality in biomedical and pharmaceutical research. Nature. (2011) 477:54–60. doi: 10.1038/nature10354 21886157 PMC3832838

[36] Lin L Huang Z Gao Y Yan X Xing J Hang W. LC-MS based serum metabonomic analysis for renal cell carcinoma diagnosis, staging, and biomarker discovery. J Proteome Res. (2011) 10:1396–405. doi: 10.1021/pr101161u 21186845

[37] Tomita I Kume S Sugahara S Osawa N Yamahara K Yasuda-Yamahara M. SGLT2 inhibition mediates protection from diabetic kidney disease by promoting ketone body-induced mTORC1 inhibition. Cell Metab. (2020) 32:404–19.e6. doi: 10.1016/j.cmet.2020.06.020 32726607

[38] Chen CH Yang WC Hsiao YH Huang SC Huang YC. High homocysteine, low vitamin B-6, and increased oxidative stress are independently associated with the risk of chronic kidney disease. Nutrition. (2016) 32:236–41. doi: 10.1016/j.nut.2015.08.016 26526964

[39] De Brandt J Derave W Vandenabeele F Pomiès P Blancquaert L Keytsman C. Efficacy of 12 weeks oral beta-alanine supplementation in patients with chronic obstructive pulmonary disease: a double-blind, randomized, placebo-controlled trial. J Cachexia Sarcopenia Muscle. (2022) 13:2361–72. doi: 10.1002/jcsm.13048 PMC9530565 35977911

[40] Yang M Vousden KH. Serine and one-carbon metabolism in cancer. Nat Rev Cancer. (2016) 16:650–62. doi: 10.1038/nrc.2016.81 27634448

[41] Agoro R White KE. Regulation of FGF23 production and phosphate metabolism by bone-kidney interactions. Nat Rev Nephrol. (2023) 19:185–93. doi: 10.1038/s41581-022-00665-x 36624273

[42] Xu Y Labedan B Glansdorff N. Surprising arginine biosynthesis: a reappraisal of the enzymology and evolution of the pathway in microorganisms. Microbiol Mol Biol Rev. (2007) 71:36–47. doi: 10.1128/MMBR.00032-06 17347518 PMC1847373

[43] Momma TY Ottaviani JI. Arginase inhibitor, N(ω)-hydroxy-L-norarginine, spontaneously releases biologically active NO-like molecule: Limitations for research applications. Free Radic Biol Med. (2020) 152:74–82. doi: 10.1016/j.freeradbiomed.2020.02.033 32131024

[44] Ikeda H. The effect of mild renal dysfunction on the assessment of plasma amino acid concentration and insulin resistance in patients with type 2 diabetes mellitus. J Diabetes Res. (2022) 2022:2048300. doi: 10.1155/2022/2048300 35734236 PMC9208954

[45] Sizeland PC Chambers ST Lever M Bason LM Robson RA. Organic osmolytes in human and other mammalian kidneys. Kidney Int. (1993) 43:448–53. doi: 10.1038/ki.1993.66 8441242

[46] Perroud B Lee J Valkova N Dhirapong A Lin PY Fiehn O. Pathway analysis of kidney cancer using proteomics and metabolic profiling. Mol Cancer. (2006) 5:64. doi: 10.1186/1476-4598-5-64 17123452 PMC1665458

[47] Fang Y Chen B Gong AY Malhotra DK Gupta R Dworkin LD. The ketone body β-hydroxybutyrate mitigates the senescence response of glomerular podocytes to diabetic insults. Kidney Int. (2021) 100:1037–53. doi: 10.1016/j.kint.2021.06.031 PMC8889914 34246657

[48] Ceballos I Chauveau P Guerin V Bardet J Parvy P Kamoun P. Early alterations of plasma free amino acids in chronic renal failure. Clin Chim Acta. (1990) 188:101–8. doi: 10.1016/0009-8981(90)90154-K 2379310

[49] Bergström J Alvestrand A Fürst P. Plasma and muscle free amino acids in maintenance hemodialysis patients without protein malnutrition. Kidney Int. (1990) 38:108–14. doi: 10.1038/ki.1990.174 2117095

[50] Alves I Santos-Pereira B Dalebout H Santos S Vicente MM Campar A. Protein mannosylation as a diagnostic and prognostic biomarker of lupus nephritis: an unusual glycan neoepitope in systemic lupus erythematosus. Arthritis Rheumatol. (2021) 73:2069–77. doi: 10.1002/art.41768 33881228

[51] Raina R Bedoyan JK Lichter-Konecki U Jouvet P Picca S Mew NA. Consensus guidelines for management of hyperammonaemia in paediatric patients receiving continuous kidney replacement therapy. Nat Rev Nephrol. (2020) 16:471–82. doi: 10.1038/s41581-020-0267-8 PMC7366888 32269302

[52] Häberle J Burlina A Chakrapani A Dixon M Karall D Lindner M. Suggested guidelines for the diagnosis and management of urea cycle disorders: First revision. J Inherit Metab Dis. (2019) 42:1192–230. doi: 10.1002/jimd.12100 30982989

[53] Lin BM Zhang Y Yu B Boerwinkle E Thygarajan B Yunes M. Metabolome-wide association study of estimated glomerular filtration rates in Hispanics. Kidney Int. (2022) 101:144–51. doi: 10.1016/j.kint.2021.09.032 PMC8741745 34774559

[54] Luo S Coresh J Tin A Rebholz CM Appel LJ Chen J. Serum metabolomic alterations associated with proteinuria in CKD. Clin J Am Soc Nephrol. (2019) 14:342–53. doi: 10.2215/CJN.10010818 PMC6419293 30733224

[55] Sekula P Goek ON Quaye L Barrios C Levey AS Römisch-Margl W. A metabolome-wide association study of kidney function and disease in the general population. J Am Soc Nephrol. (2016) 27:1175–88. doi: 10.1681/ASN.2014111099 PMC4814172 26449609

[56] Gaudry S Palevsky PM Dreyfuss D. Extracorporeal kidney-replacement therapy for acute kidney injury. N Engl J Med. (2022) 386:964–75. doi: 10.1056/NEJMra2104090 35263520

[57] James MT Bhatt M Pannu N Tonelli M. Long-term outcomes of acute kidney injury and strategies for improved care. Nat Rev Nephrol. (2020) 16:193–205. doi: 10.1038/s41581-019-0247-z 32051567

[58] Fujimoto T Hyodo Y Ishimura T Tashiro Y Endo T Nisioka S. Association of alpha-actinin-3 polymorphism with sarcopenia in kidney transplant recipients. Transplant Proc. (2023) 55:824–8. doi: 10.1016/j.transproceed.2023.03.020 37037724

[59] Jin H Yoo HJ Kim YA Lee JH Lee Y Kwon SH. Unveiling genetic variants for age-related sarcopenia by conducting a genome-wide association study on Korean cohorts. Sci Rep. (2022) 12:3501. doi: 10.1038/s41598-022-07567-9 35241739 PMC8894365

[60] Fernández-Lázaro D Hernández JLG Lumbreras E Mielgo-Ayuso J Seco-Calvo J. 25-Hydroxyvitamin D Serum Levels Linked to Single Nucleotide Polymorphisms (SNPs) (rs2228570, rs2282679, rs10741657) in Skeletal Muscle Aging in Institutionalized Elderly Men Not Supplemented with Vitamin D. Int J Mol Sci. (2022) 23:11846. doi: 10.3390/ijms231911846 36233147 PMC9569711

[61] Lin CH Lin CC Tsai CW Chang WS Yang MD Bau DT. A novel caveolin-1 biomarker for clinical outcome of sarcopenia. In Vivo. (2014) 28:383–9. 24815842

[62] Liu Z Chaillou T Santos Alves E Mader T Jude B Ferreira DMS. Mitochondrial NDUFA4L2 is a novel regulator of skeletal muscle mass and force. FASEB J. (2021) 35:e22010. doi: 10.1096/fj.202100066R 34724256

[63] Okamura T Okada H Hashimoto Y Majima S Senmaru T Nakanishi N. Let-7e-5p regulates IGF2BP2, and induces muscle atrophy. Front Endocrinol (Lausanne). (2021) 12:791363. doi: 10.3389/fendo.2021.791363 35002969 PMC8741024

[64] Han DS Wu WK Liu PY Yang YT Hsu HC Kuo CH. Differences in the gut microbiome and reduced fecal butyrate in elders with low skeletal muscle mass. Clin Nutr. (2022) 41:1491–500. doi: 10.1016/j.clnu.2022.05.008 35667265

