# Supplementary material for: Causal effects of plasma metabolites on chronic kidney diseases and renal function: a bidirectional Mendelian randomization study
Source: Front Endocrinol (Lausanne). 2024 Jul 26;15:1429159. doi: 10.3389/fendo.2024.1429159 (PMC11310041; doi:10.3389/fendo.2024.1429159)
Supplement: Supplementary file 1 [file DataSheet_1.docx]

**Supplementary Figures**

**Contents**

**Supplementary Figure S1.** Scatter plots of the genetic association of five plasma metabolites or metabolite ratios on the risk of chronic kidney disease.

**Supplementary Figure S2.** Leave-one-out sensitivity analyses of the SNPs represented the five plasma metabolites or metabolite ratios and chronic kidney disease.

**Supplementary Figure S3.** Forest plots for five plasma metabolites or metabolite ratios on chronic kidney disease.

**Supplementary Figure S4.** Scatter plot for estimating the risk of fourteen plasma metabolites or metabolite ratios on the change in creatinine-eGFR.

**Supplementary Figure S5**. Leave-one-out sensitivity analyses of the SNPs represented the fourteen plasma metabolites or metabolite ratios and creatinine-eGFR.

**Supplementary Figure S6**. Forest plots for fourteen plasma metabolites or metabolite ratios on creatinine-eGFR.

**Supplementary Figure S7.** Scatter plot for estimating the risk of seven plasma metabolites or metabolite ratios on the change in urine albumin creatine ratio.

**Supplementary Figure S8.** Leave-one-out sensitivity analyses of the SNPs represented the seven plasma metabolites or metabolite ratios and urine albumin creatine ratio.

**Supplementary Figure S9.** Forest plots for seven plasma metabolites or metabolite ratios on urine albumin creatine ratio.

**Supplementary Figure S10**. Regional association plots for colocalization analysis of six plasma metabolites or metabolite ratios with the risk of chronic kidney disease.

**Supplementary Figure S11.** Regional association plots for colocalization analysis of sixteen plasma metabolites or metabolite ratios with the risk of creatinine-eGFR.

**Supplementary Figure S12.** Regional association plots for colocalization analysis of seven plasma metabolites or metabolite ratios with the risk of urine albumin creatine ratio.


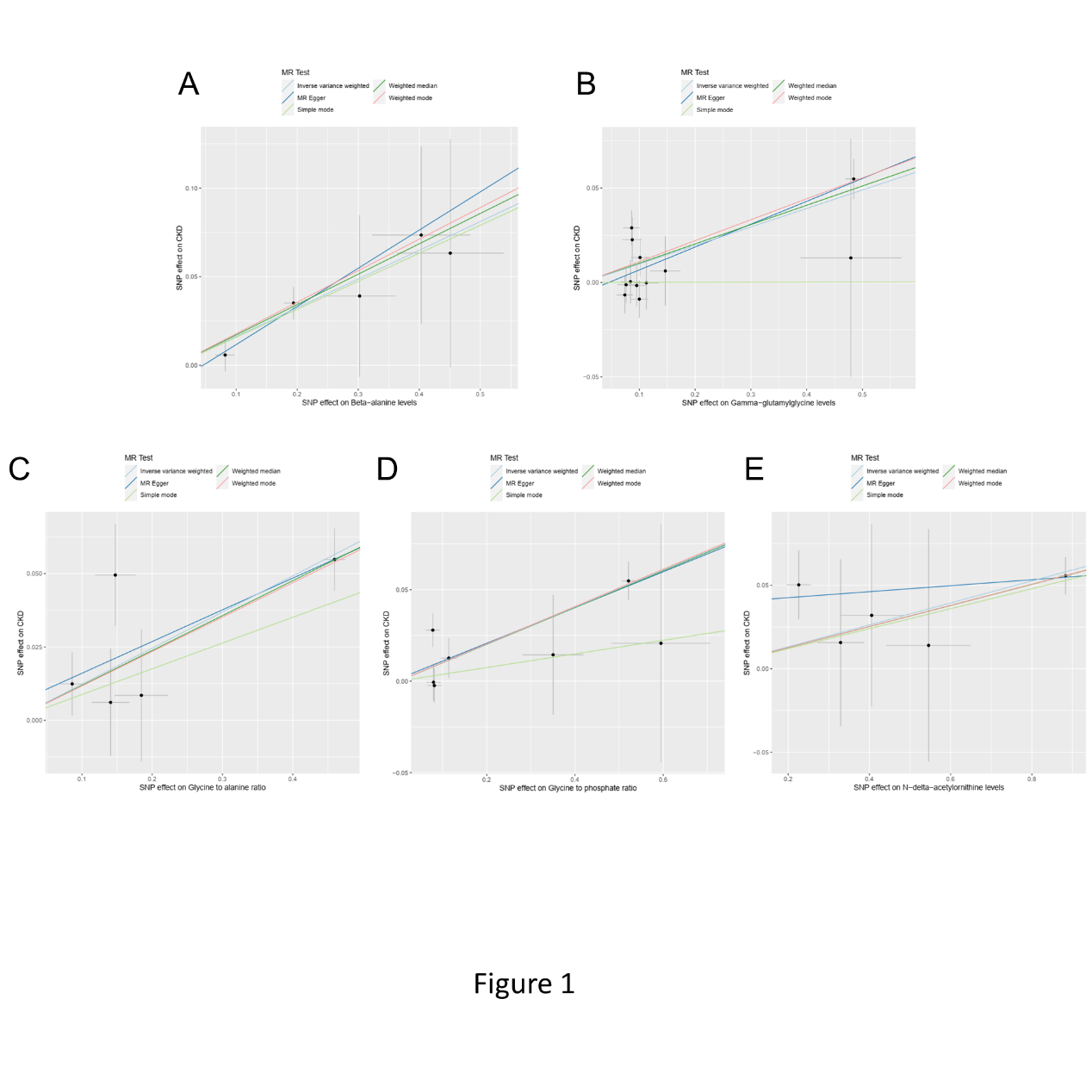


**Supplementary Figure S1. Scatter plots of** **the genetic association of five plasma metabolites or metabolite ratios on the risk of chronic kidney disease.** Beta-alanine levels (A), Gamma-glutamylglycine levels (B), Glycine to alanine ratio (C), Glycine to phosphate ratio (D), N-delta-acetylornithine levels (E). Each black point representing a SNP is plotted in relation to the effect size of the SNP on the exposure (x-axis) and on the outcome (y-axis) with corresponding standard error bars. The slope of each line corresponds to the causal estimate using IVW (light blue), MR-Egger regression (blue), Simple mode (light green), weighted median (green), and weighted mode (pink) method.


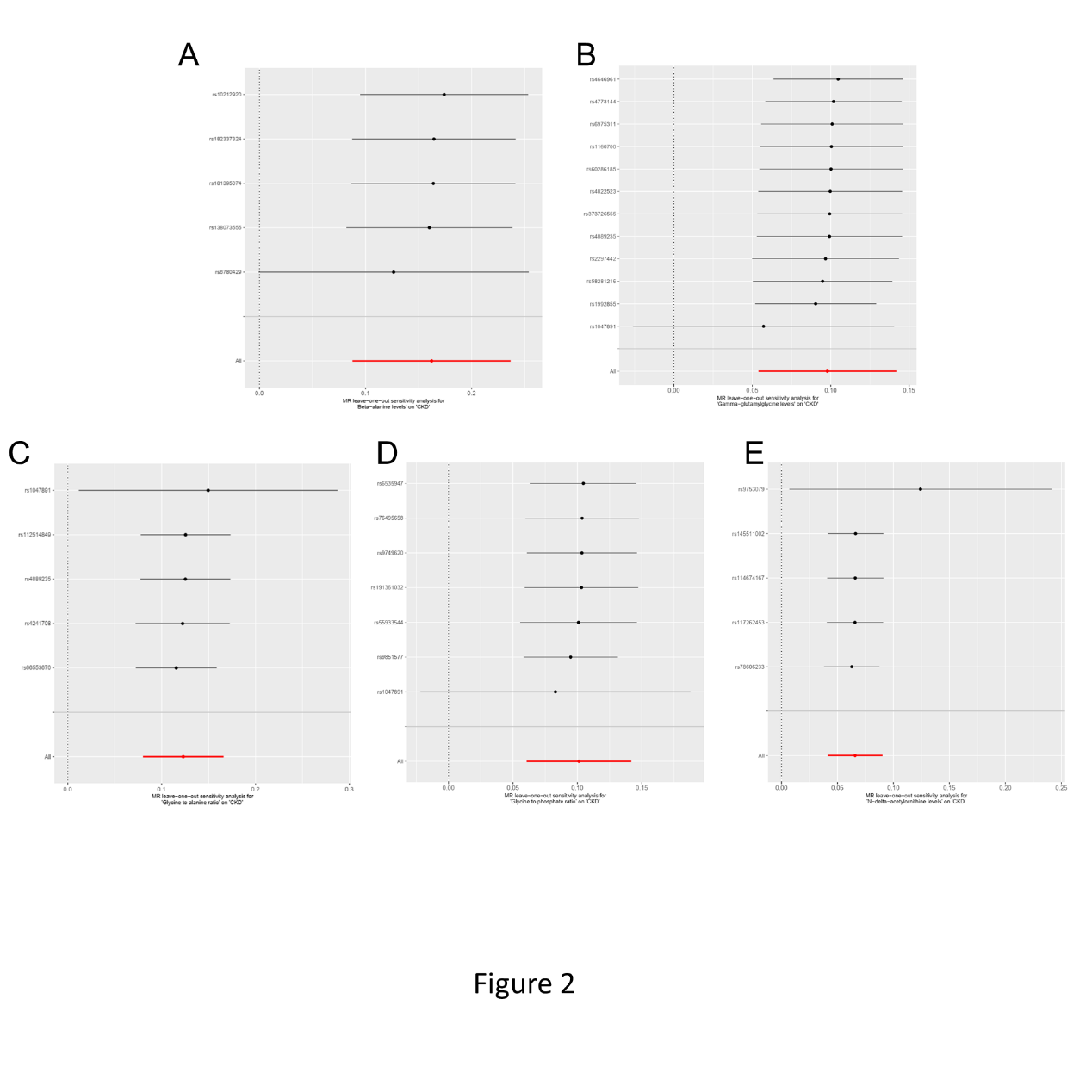


**Supplementary Figure S2. Leave-one-out sensitivity analyses of the SNPs represented the five plasma metabolites or metabolite ratios and chronic kidney disease.** Beta-alanine levels (A), Gamma-glutamylglycine levels (B), Glycine to alanine ratio (C), Glycine to phosphate ratio (D), N-delta-acetylornithine levels (E). The estimated causal effect is shown for each excluded SNP and the overall estimate using all the SNPs is shown in red. The error bars represent the 95% confidence intervals.


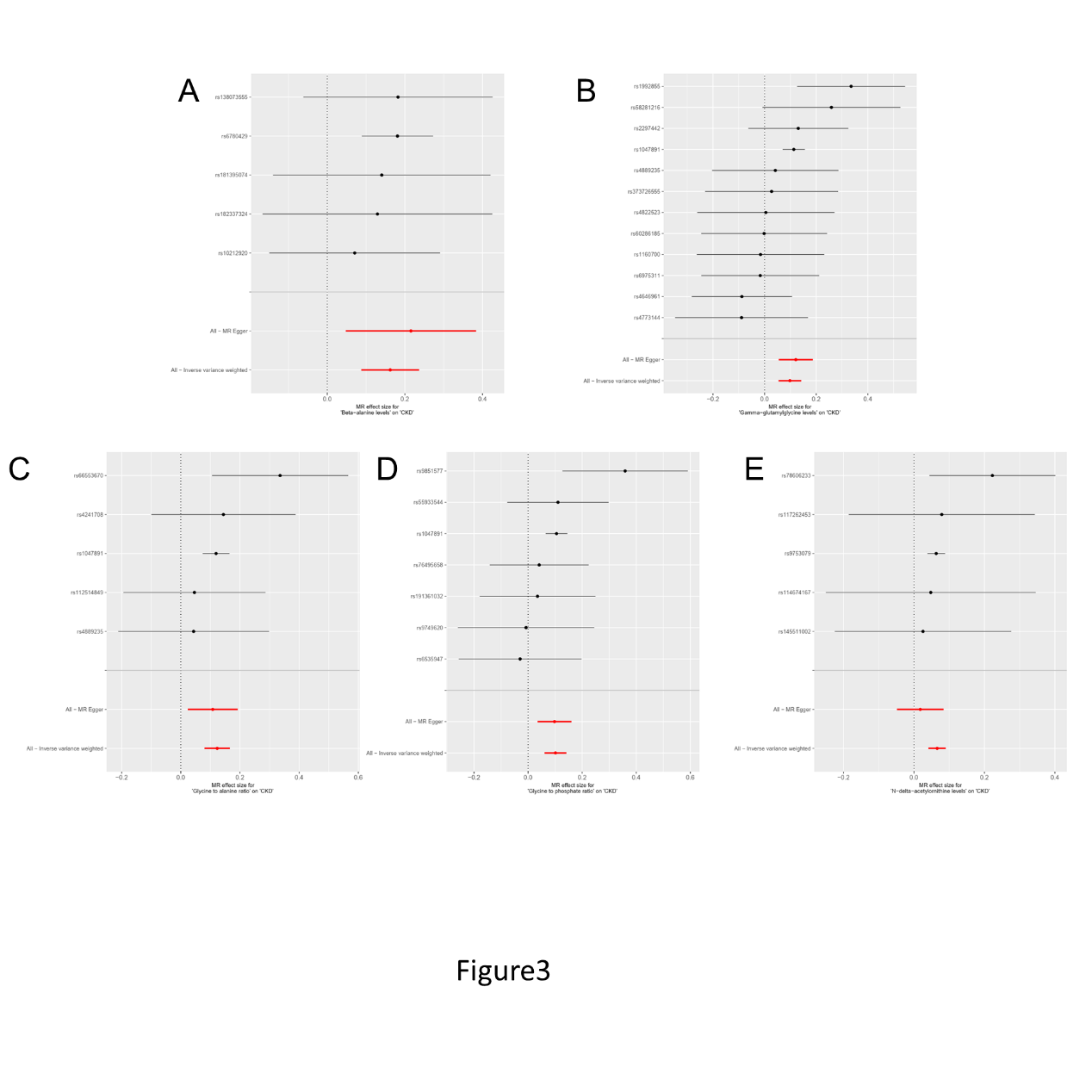


**Supplementary Figure S3. Forest plots for five plasma metabolites or metabolite ratios on chronic kidney disease.** Beta-alanine levels (A), Gamma-glutamylglycine levels (B), Glycine to alanine ratio (C), Glycine to phosphate ratio (D), N-delta-acetylornithine levels (E). Each horizontal solid line reflects the estimated result of a single SNP using the Wald ratio method. The bottom two red lines reflect the overall estimate using all SNPs under the IVW and MR-Egger methods. The red line is completely to the left of 0, indicating a significant negative correlation between exposure factors and outcome. The red line is completely to the right of 0, indicating a significant positive correlation between exposure factors and outcome. If the red line crosses 0, the result is not significant.


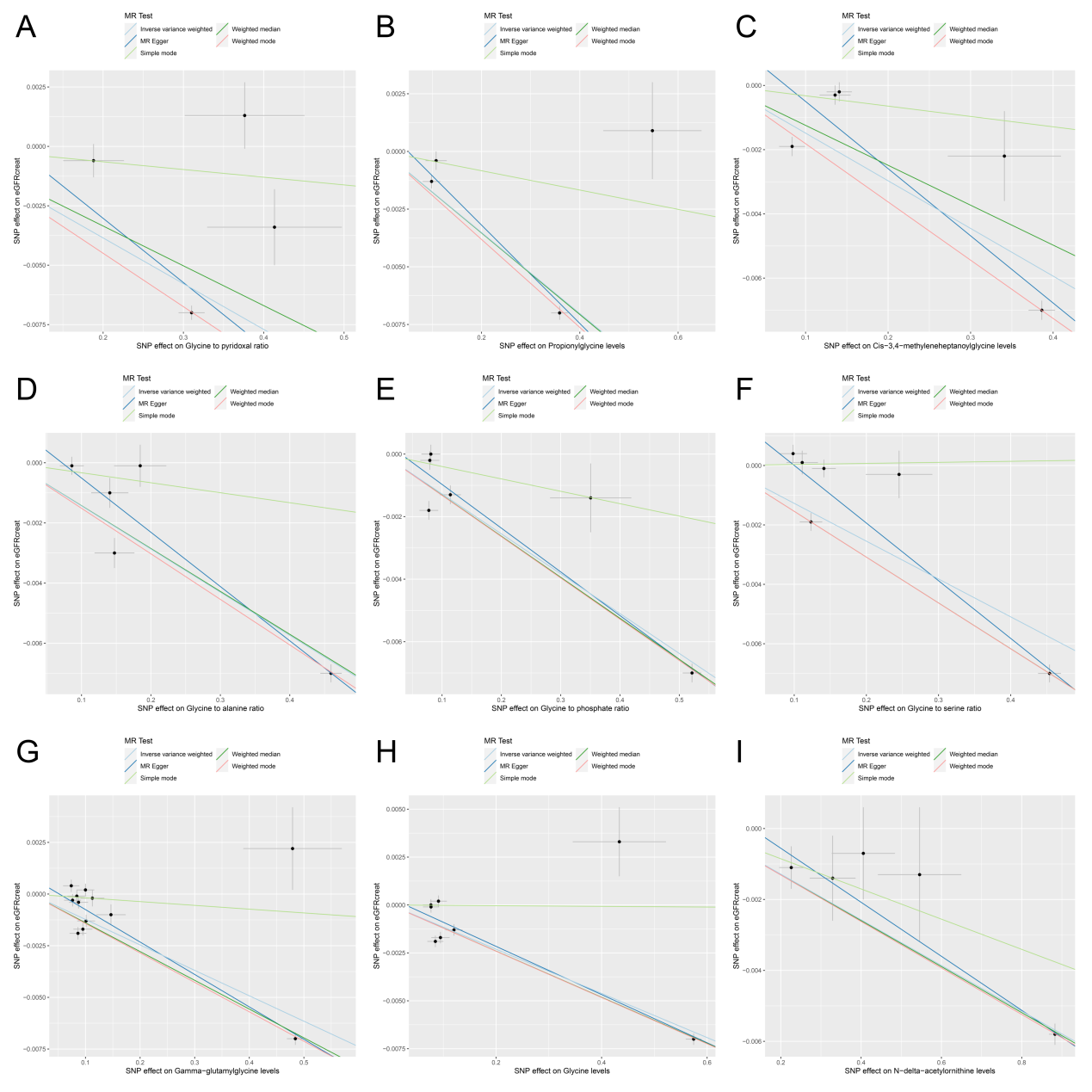


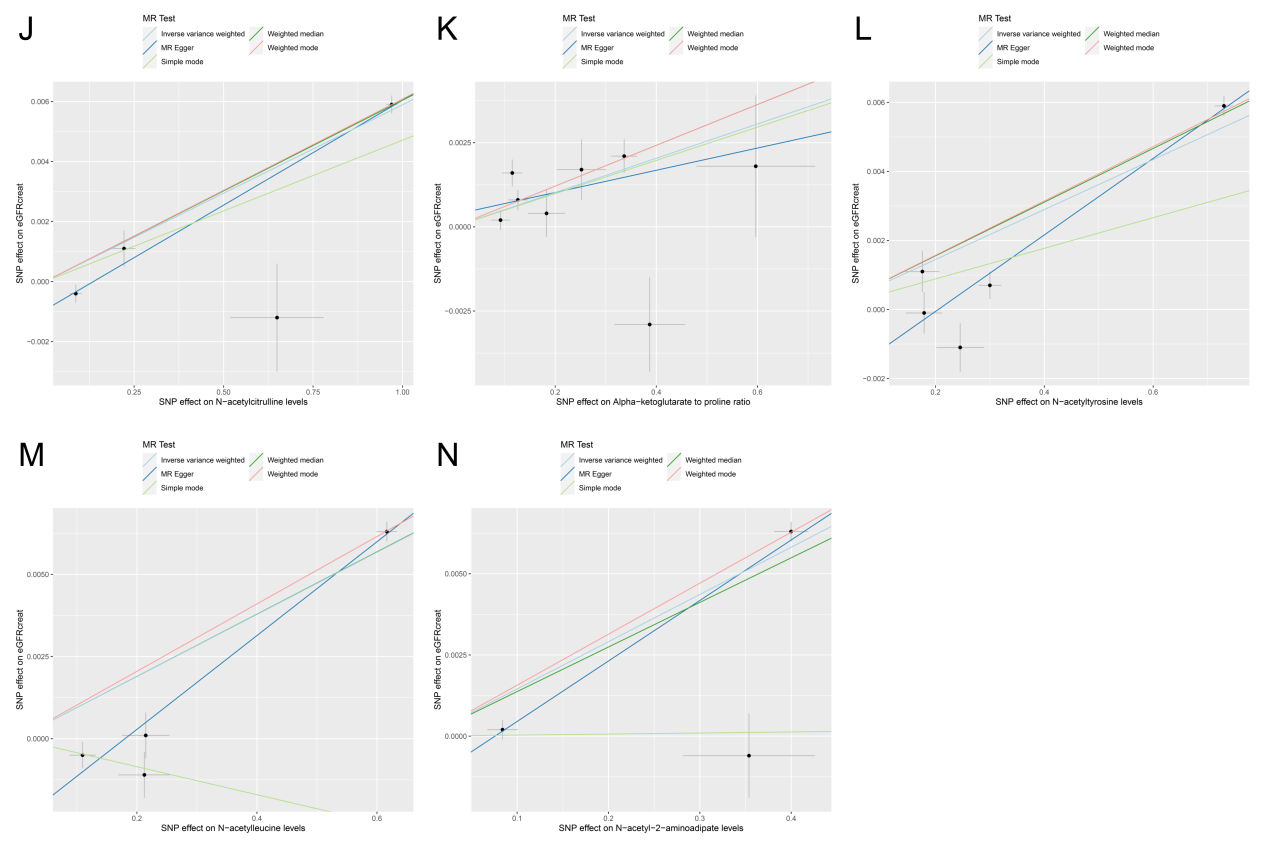


**Supplementary Figure S4. Scatter plot for estimating the risk of fourteen plasma metabolites or metabolite ratios on the change in creatinine-eGFR.** Glycine to pyridoxal ratio (A), Propionylglycine levels (B), Cis-3,4-methyleneheptanoylglycine levels (C), Glycine to alanine ratio (D), Glycine to phosphate ratio (E), Glycine to serine ratio (F), Gamma-glutamylglycine levels (G), Glycine levels (H), N-delta-acetylornithine levels (I), N-acetylcitrulline levels (J), Alpha-ketoglutarate to proline ratio (K), N-acetyltyrosine levels (L), N-acetylleucine levels (M), N-acetyl-2-aminoadipate levels (N). Each black point representing a SNP is plotted in relation to the effect size of the SNP on the exposure (x-axis) and on the outcome (y-axis) with corresponding standard error bars. The slope of each line corresponds to the causal estimate using IVW (light blue), MR-Egger regression (blue), Simple mode (light green), weighted median (green), and weighted mode (pink) method.


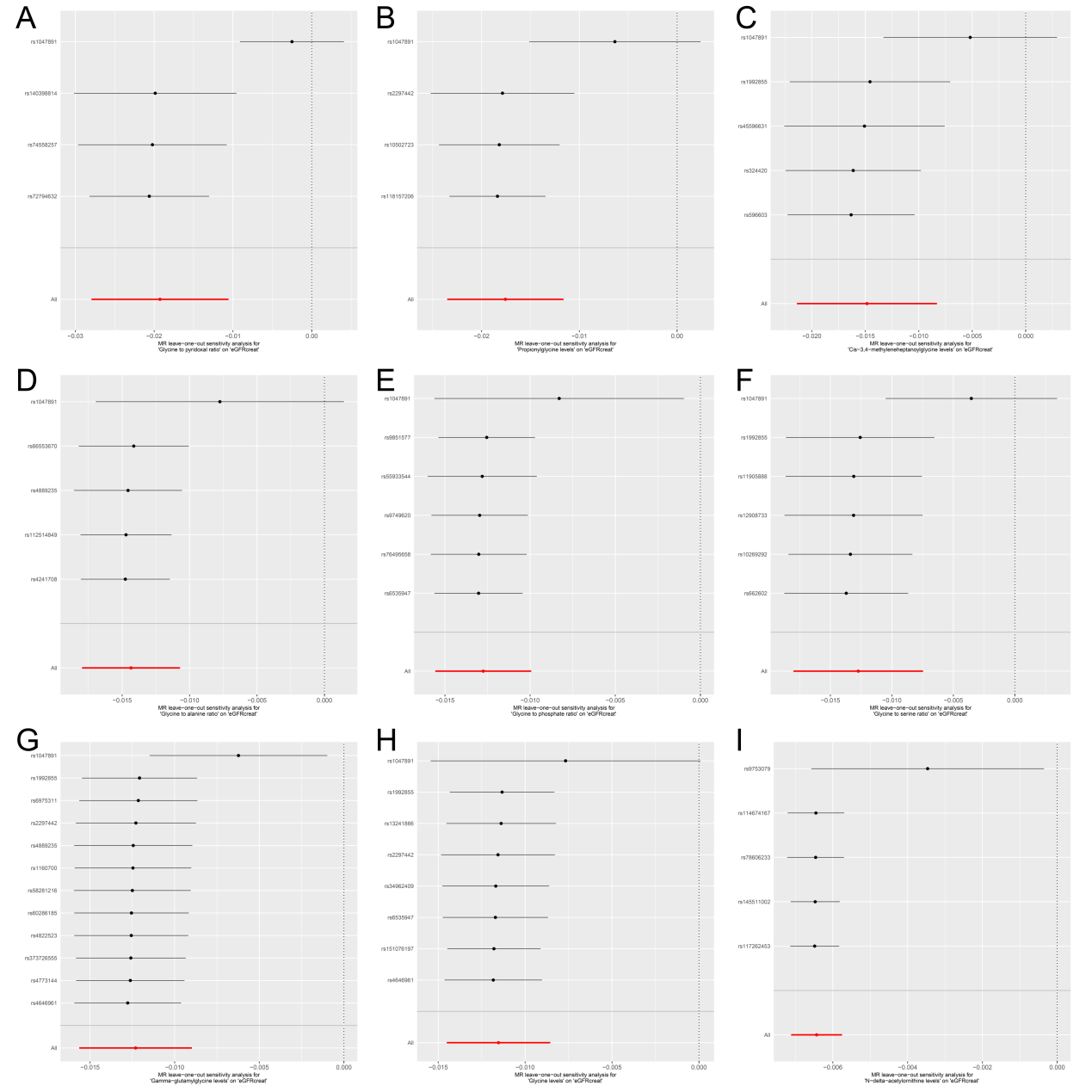


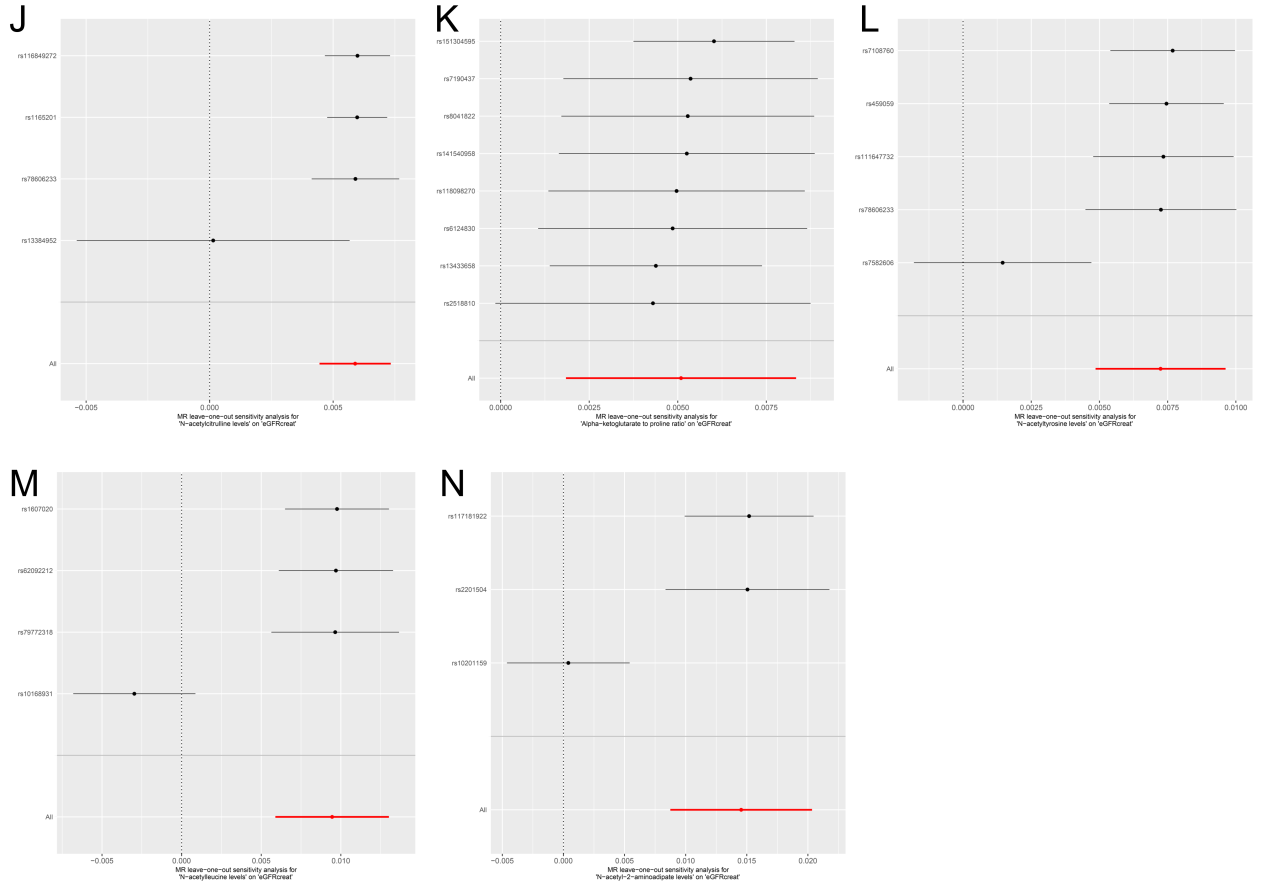


**Supplementary Figure S5. Leave-one-out sensitivity analyses of the SNPs represented the fourteen plasma metabolites or metabolite ratios and creatinine-eGFR.** Glycine to pyridoxal ratio (A), Propionylglycine levels (B), Cis-3,4-methyleneheptanoylglycine levels (C), Glycine to alanine ratio (D), Glycine to phosphate ratio (E), Glycine to serine ratio (F), Gamma-glutamylglycine levels (G), Glycine levels (H), N-delta-acetylornithine levels (I), N-acetylcitrulline levels (J), Alpha-ketoglutarate to proline ratio (K), N-acetyltyrosine levels (L), N-acetylleucine levels (M), N-acetyl-2-aminoadipate levels (N). The estimated causal effect is shown for each excluded SNP and the overall estimate using all the SNPs is shown in red. The error bars represent the 95% confidence intervals.


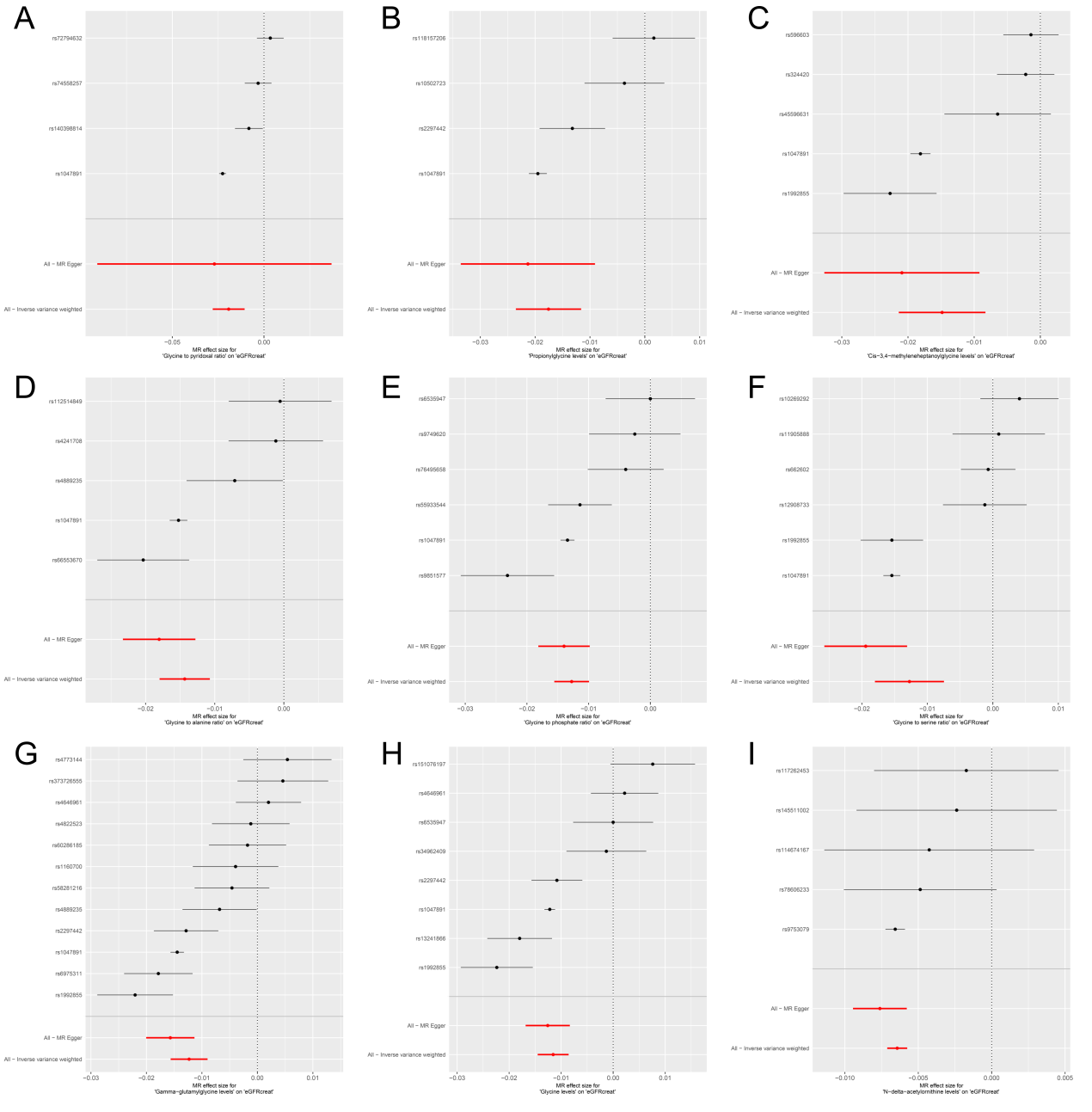


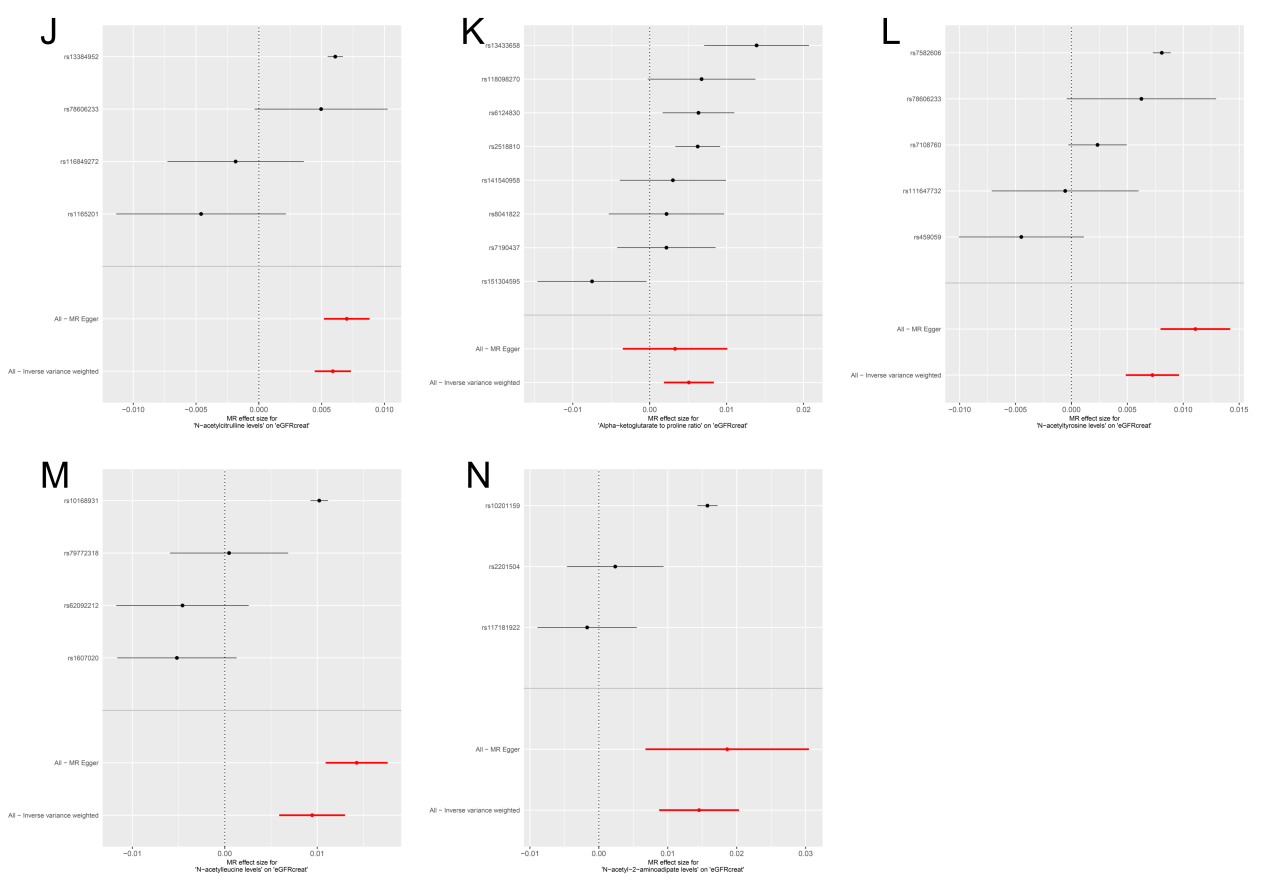


**Supplementary Figure S6.** **Forest plots for fourteen plasma metabolites or metabolite ratios on creatinine-eGFR.** Glycine to pyridoxal ratio (A), Propionylglycine levels (B), Cis-3,4-methyleneheptanoylglycine levels (C), Glycine to alanine ratio (D), Glycine to phosphate ratio (E), Glycine to serine ratio (F), Gamma-glutamylglycine levels (G), Glycine levels (H), N-delta-acetylornithine levels (I), N-acetylcitrulline levels (J), Alpha-ketoglutarate to proline ratio (K), N-acetyltyrosine levels (L), N-acetylleucine levels (M), N-acetyl-2-aminoadipate levels (N). Each horizontal solid line reflects the estimated result of a single SNP using the Wald ratio method. The bottom two red lines reflect the overall estimate using all SNPs under the IVW and MR-Egger methods. The red line is completely to the left of 0, indicating a significant negative correlation between exposure factors and outcome. The red line is completely to the right of 0, indicating a significant positive correlation between exposure factors and outcome. If the red line crosses 0, the result is not significant.


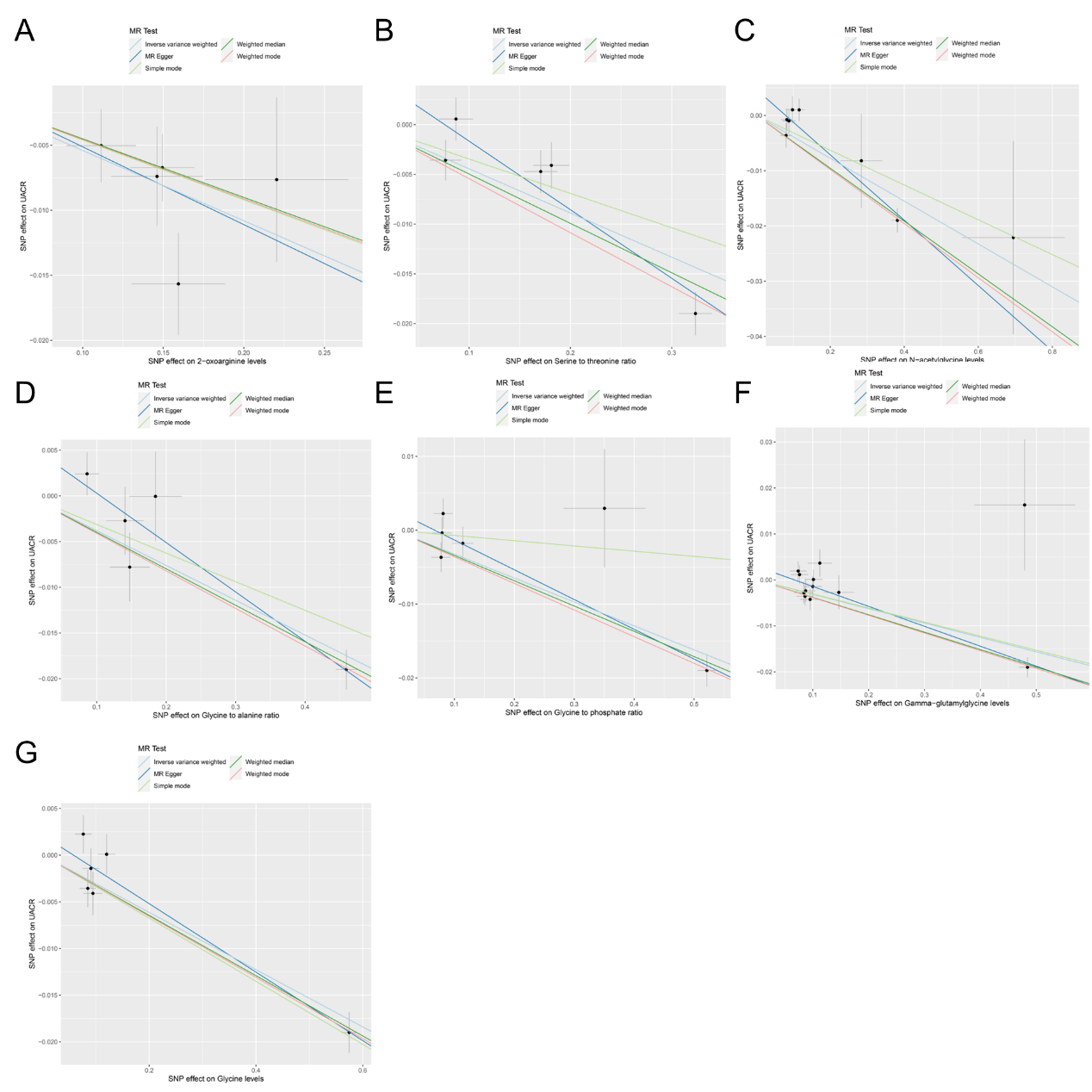


**Supplementary Figure S7. Scatter plot for estimating the risk of seven plasma metabolites or metabolite ratios on the change in urine albumin creatine ratio.** 2-oxoarginine levels (A), Serine to threonine ratio (B), N-acetylglycine levels (C), Glycine to alanine ratio (D), Glycine to phosphate ratio (E), Gamma-glutamylglycine levels (F), Glycine levels (G). Each black point representing a SNP is plotted in relation to the effect size of the SNP on the exposure (x-axis) and on the outcome (y-axis) with corresponding standard error bars. The slope of each line corresponds to the causal estimate using IVW (light blue), MR-Egger regression (blue), Simple mode (light green), weighted median (green), and weighted mode (pink) method.


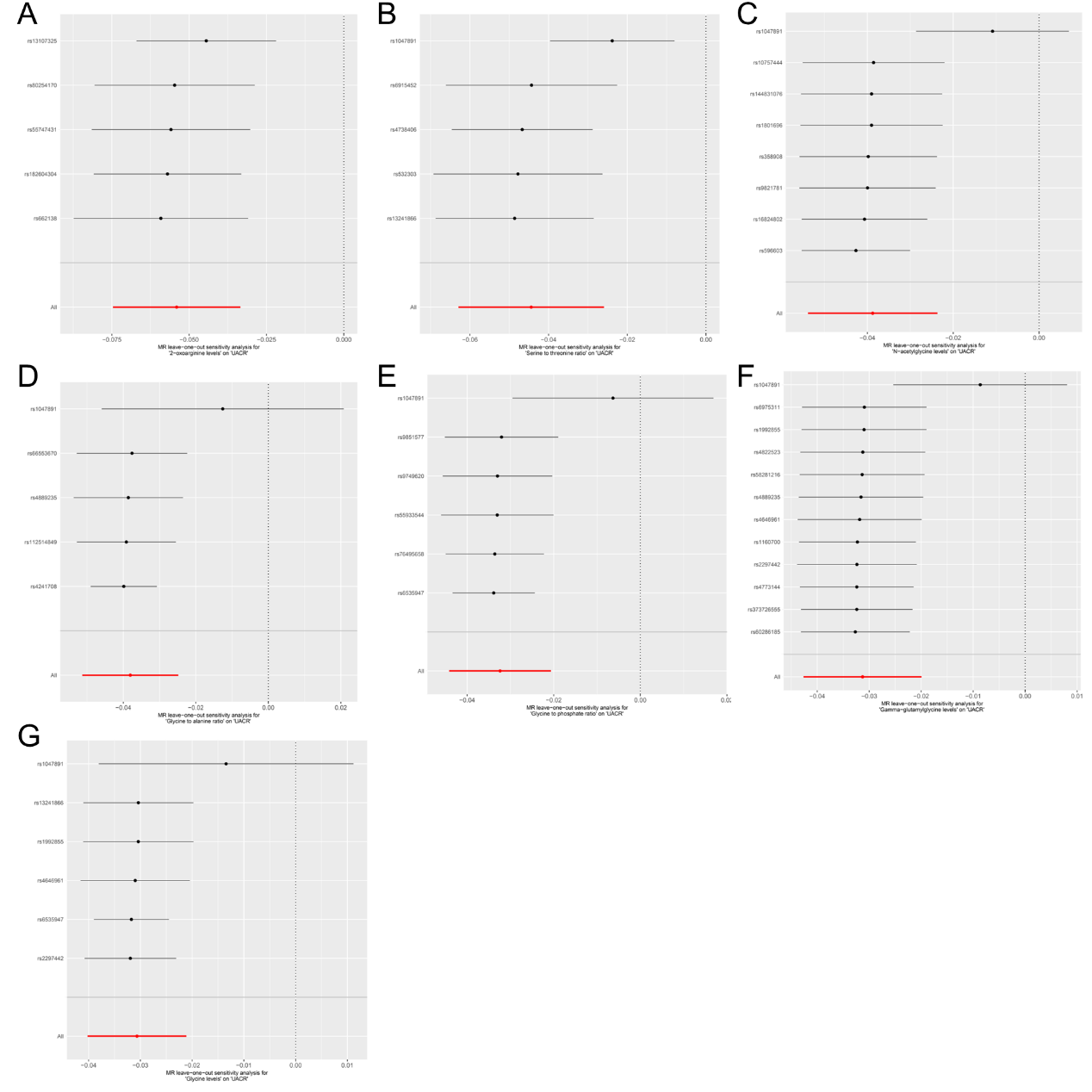


**Supplementary Figure S8. Leave-one-out sensitivity analyses of the SNPs represented the seven plasma metabolites or metabolite ratios and urine albumin creatine ratio.** 2-oxoarginine levels (A), Serine to threonine ratio (B), N-acetylglycine levels (C), Glycine to alanine ratio (D), Glycine to phosphate ratio (E), Gamma-glutamylglycine levels (F), Glycine levels (G). The estimated causal effect is shown for each excluded SNP and the overall estimate using all the SNPs is shown in red. The error bars represent the 95% confidence intervals.


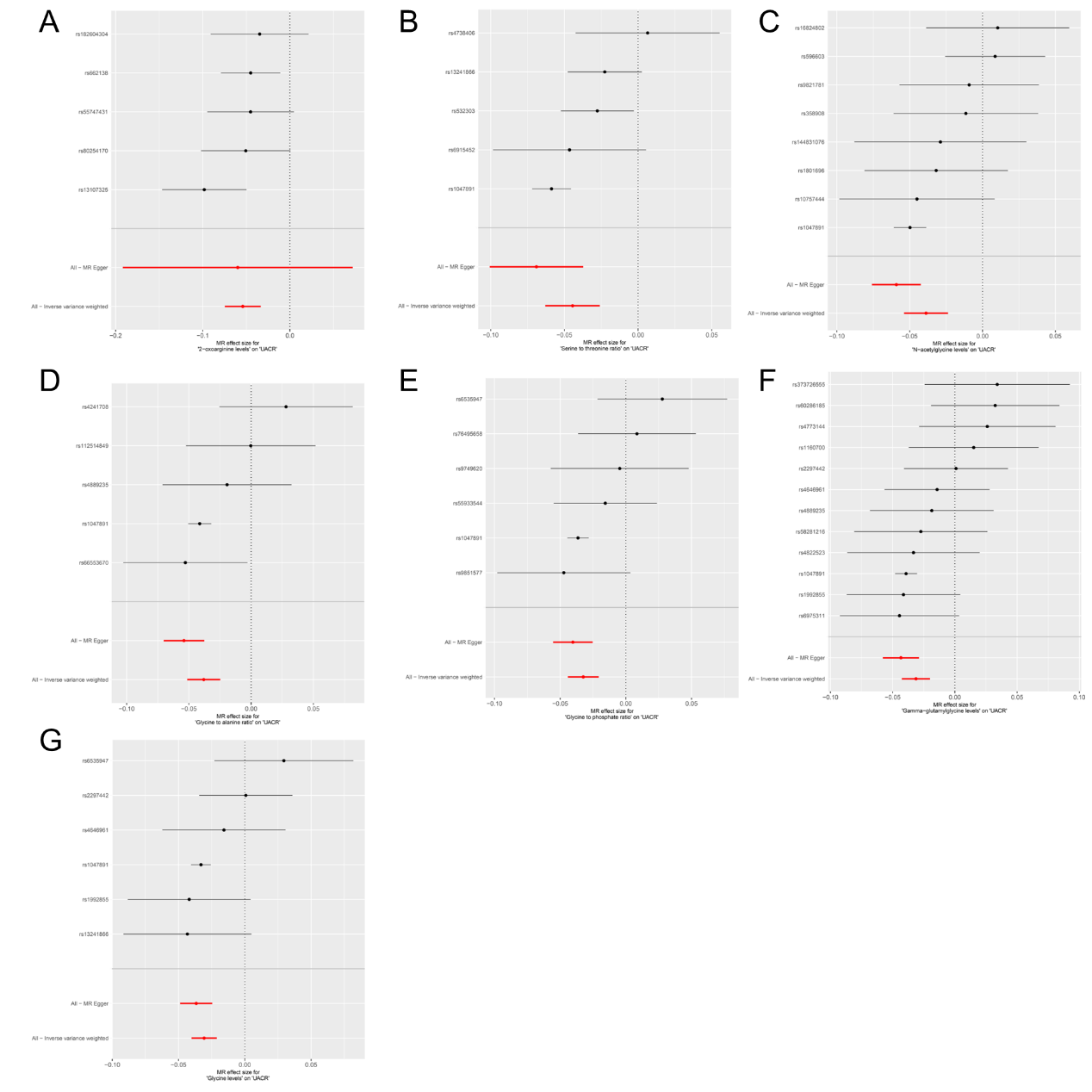


**Supplementary Figure S9.** **Forest plots for seven plasma metabolites or metabolite ratios on urine albumin creatine ratio.** 2-oxoarginine levels (A), Serine to threonine ratio (B), N-acetylglycine levels (C), Glycine to alanine ratio (D), Glycine to phosphate ratio (E), Gamma-glutamylglycine levels (F), Glycine levels (G). Each horizontal solid line reflects the estimated result of a single SNP using the Wald ratio method. The bottom two red lines reflect the overall estimate using all SNPs under the IVW and MR-Egger methods. The red line is completely to the left of 0, indicating a significant negative correlation between exposure factors and outcome. The red line is completely to the right of 0, indicating a significant positive correlation between exposure factors and outcome. If the red line crosses 0, the result is not significant.


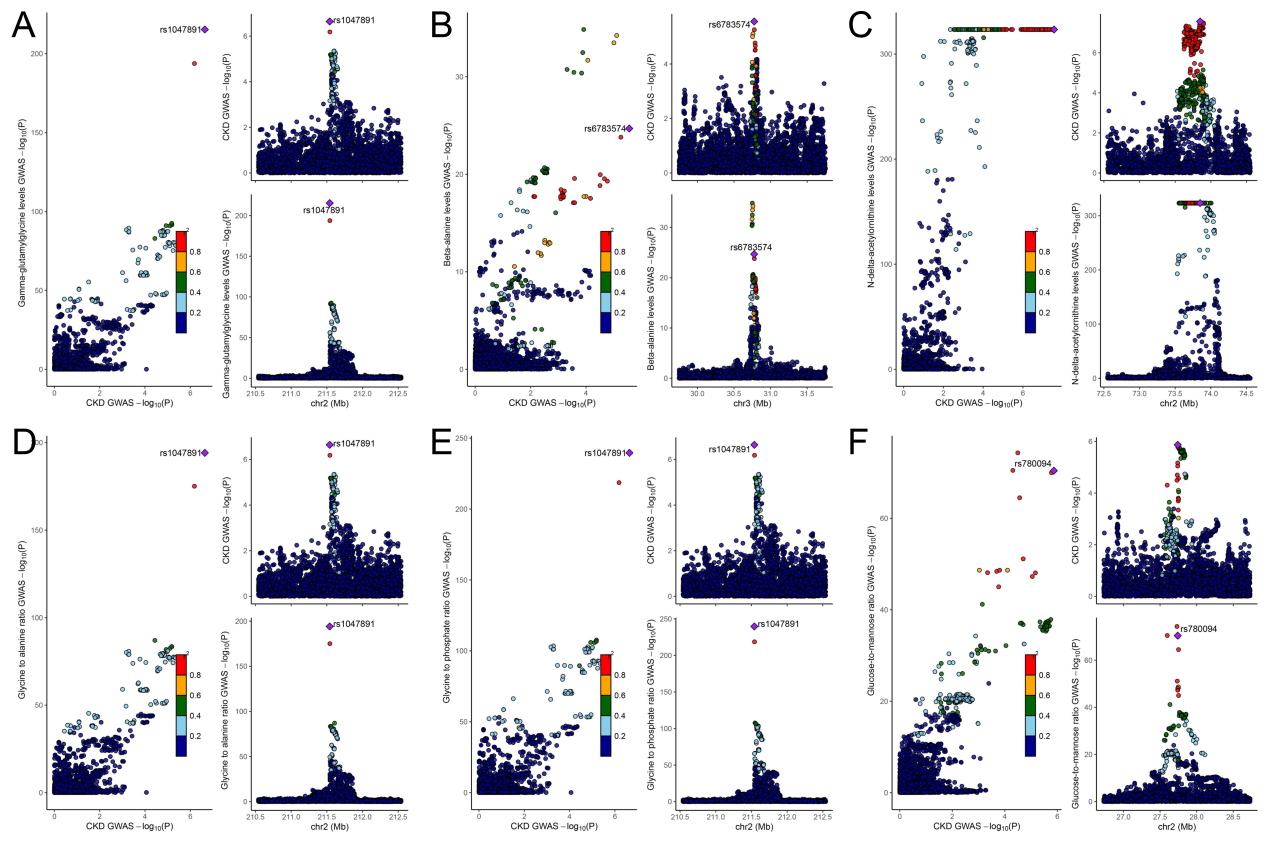


**Supplementary Figure S10. Regional association plots for colocalization analysis of six plasma metabolites or metabolite ratios with the risk of chronic kidney disease.** Gamma-glutamylglycine levels (A), Beta-alanine levels (B), N-delta-acetylornithine levels (C), Glycine to alanine ratio (D), Glycine to phosphate ratio (E), Glucose-to-mannose ratio (F). The lead SNP is shown as a purple diamond. SNPs within ± 1000 kb of the lead SNP were included; p12=1e-5, prior probability a SNP is associated with both metabolites/metabolite ratios and chronic kidney disease.


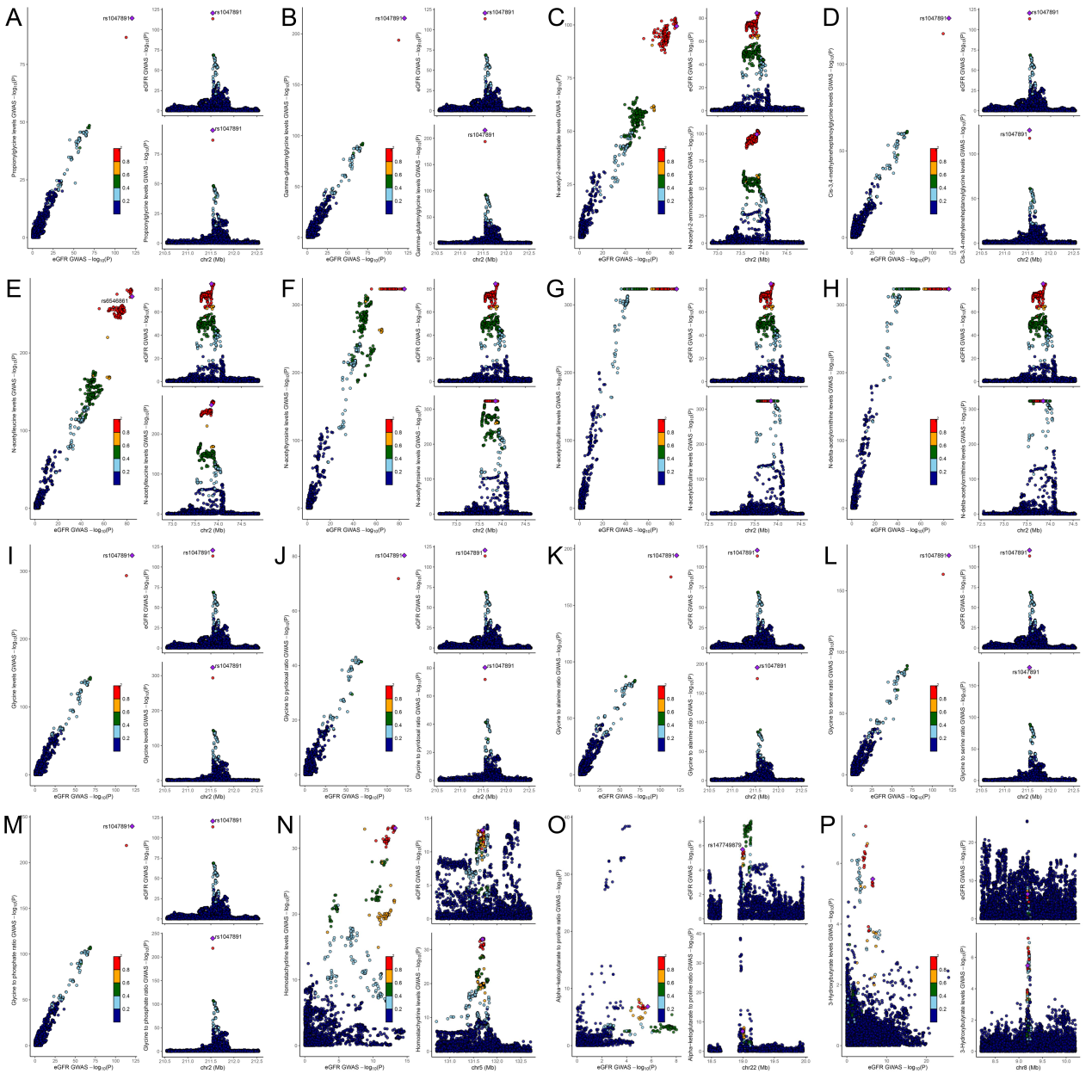


**Supplementary Figure S11. Regional association plots for colocalization analysis of sixteen plasma metabolites or metabolite ratios with the risk of creatinine-eGFR.** Propionylglycine levels (A), Gamma-glutamylglycine levels (B), N-acetyl-2-aminoadipate levels (C), Cis-3,4-methyleneheptanoylglycine levels (D), N-acetylleucine levels (E), N-acetyltyrosine levels (F), N-acetylcitrulline levels (G), N-delta-acetylornithine levels (H), Glycine levels (I), Glycine to pyridoxal ratio (J), Glycine to alanine ratio (K), Glycine to serine ratio (L), Glycine to phosphate ratio (M), Homostachydrine levels (N), Alpha-ketoglutarate to proline ratio (O), 3-Hydroxybutyrate levels (P). The lead SNP is shown as a purple diamond. SNPs within ± 1000 kb of the lead SNP were included; p12=1e-5, prior probability a SNP is associated with both metabolites/metabolite ratios and estimated glomerular filtration rate.


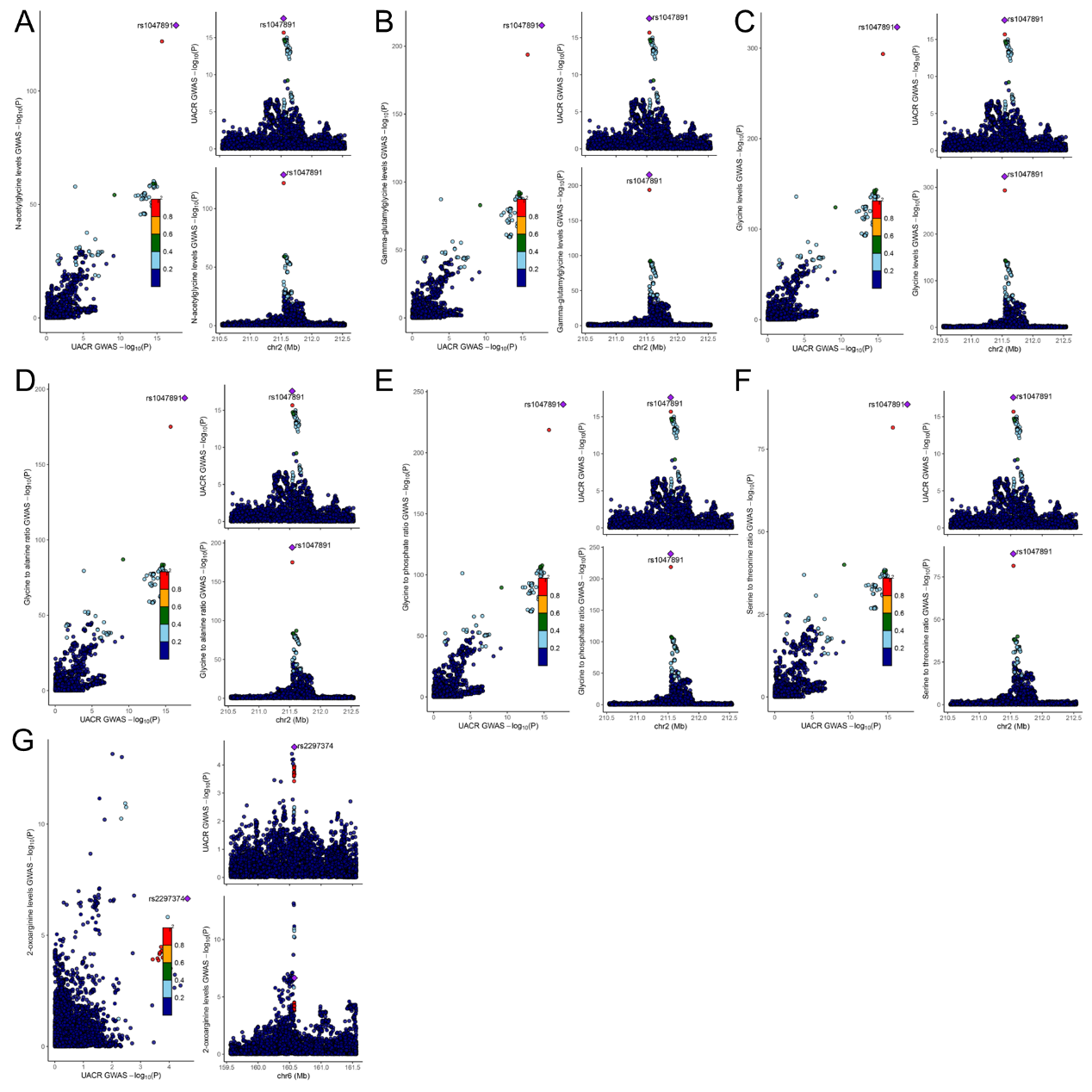


**Supplementary Figure S12.** **Regional association plots for colocalization analysis of seven plasma metabolites or metabolite ratios with the risk of urine albumin creatine ratio.** N-acetylglycine levels (A), Gamma-glutamylglycine levels (B), Glycine levels (C), Glycine to alanine ratio (D), Glycine to phosphate ratio (E), Serine to threonine ratio (F), 2-oxoarginine levels (G). The lead SNP is shown as a purple diamond. SNPs within ± 1000 kb of the lead SNP were included; p12=1e-5, prior probability a SNP is associated with both metabolites/metabolite ratios and urine albumin creatine ratio.

Figure 14. Colocalization analysis of plasma metabolites with eGFR. Figure A to Figure M successively show the colocalization analysis of Propionylglycine levels, Gamma-glutamylglycine levels, N-acetyl-2-aminoadipate levels, Cis-3,4-methyleneheptanoylglycine levels, N-acetylleucine levels, N-acetyltyrosine levels, , N-acetylcitrulline levels, N-delta-acetylornithine levels, Glycine levels, Glycine to pyridoxal ratio, Glycine to alanine ratio, Glycine to serine ratio, Glycine to phosphate ratio, Homostachydrine levels, Alpha-ketoglutarate to proline ratio, 3-Hydroxybutyrate levels with eGFR.

Figure 15. Colocalization analysis of plasma metabolites with UACR. Figure A to Figure H successively show the colocalization analysis of N-acetylglycine levels，Gamma-glutamylglycine levels, Creatine levels, Creatinine levels, Glycine levels, Glycine to alanine ratio, Glycine to phosphate ratio, Serine to threonine ratio, 2-oxoarginine levels with UACR.
